# RNAi Strategies Against Downy Mildews: Insights Into dsRNA Uptake and Silencing

**DOI:** 10.1111/mpp.70140

**Published:** 2025-08-18

**Authors:** Deniz Göl, Emeka Okechukwu, Gizem Ünal, Anne Webb, Tom Wood, Yiguo Hong, Sherif M. Sherif, Theresa Wacker, David J. Studholme, John M. McDowell, Mahmut Tör

**Affiliations:** ^1^ Molecular Plant and Microbial Biosciences Research Unit, School of Science and the Environment University of Worcester Worcester UK; ^2^ NIAB Plant Pathology Department Cambridge UK; ^3^ Hebei International Research Centre of Vegetable Functional Genomics, College of Horticulture Hebei Agricultural University Baoding China; ^4^ Alson H. Smith Jr. Agricultural Research and Extension Center, School of Plant and Environmental Sciences Virginia Tech Winchester Virginia USA; ^5^ Biosciences University of Exeter Exeter UK; ^6^ School of Plant and Environmental Sciences Virginia Tech Blacksburg Virginia USA

**Keywords:** downy mildews, dsRNA‐mediated gene silencing, model and crop plants, oomycetes, plant‐microbe interactions

## Abstract

Downy mildew (DM) diseases are caused by destructive obligate pathogens with limited control options, posing a significant threat to global agriculture. RNA interference (RNAi) has emerged as a promising, environmentally sustainable strategy for disease management. We evaluated the efficacy of dsRNA‐mediated RNAi in suppressing key biological functions in DM pathogens of *Arabidopsis thaliana* , pea and lettuce: *Hyaloperonospora arabidopsidis* (*Hpa*), *Peronospora viciae* f. sp. *pisi* (*Pvp*) and *Bremia lactucae* (*Bl*), respectively. Conserved genes, *cellulose synthase 3* (*CesA3*) and *beta‐tubulin* (*BTUB*), were targeted. Silencing these genes significantly impaired spore germination and infection across species and reduced gene expression correlated with suppressed sporulation, confirming silencing efficacy. We tested dsRNAs from chemical synthesis, in vitro transcription, and *Escherichia coli* expression. Uptake and silencing efficiency varied with dsRNA length and concentration. In *Hpa*, short dsRNAs (21–25 bp) produced a variable spore germination rate, with 25 bp dsRNA causing a 247.10% increase, whereas longer dsRNAs (≥ 30 bp) completely inhibited germination. Similarly, in *Pvp*, dsRNAs of 21–25 bp resulted in a 73.05%–77.46% germination rate, while 30–75 bp dsRNAs abolished germination. Confocal microscopy using Cy‐5‐labelled short‐synthesised dsRNA (SS‐dsRNA) confirmed uptake by spores. Sequence specificity influenced efficacy, highlighting the need for precise target design. Multiplexed RNAi impacted silencing synergistically, further reducing germination and sporulation in *Hpa*. Importantly, SS‐dsRNA‐mediated silencing was durable, with reduced gene expression sustained at 4, 7, 10 and 11 days post‐inoculation. Taken together, our findings demonstrate the potential of dsRNA‐mediated gene silencing as a precise, sustainable tool for managing DM pathogens in multiple crop species.

## Graphical abstract

Use of dsRNA to target genes in downy mildews by co‐applying with inoculum. Pathogen development was assessed via visual scoring, molecular tools and microscopy. This supports gene function studies and disease control strategies.

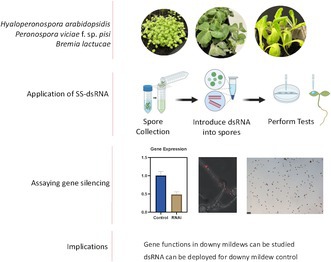

## 1 Introduction

Downy mildews diseases (DMs) are caused by a group of obligate biotrophic oomycetes and are among the most destructive plant pathogens, causing severe economic losses to crops such as grapevine, lettuce, spinach and cucurbits (Haile et al. 2021; Govindarajulu et al. 2015; Bianca et al. 2023; Tör et al. 2023). Unlike hemibiotrophic oomycetes such as *Phytophthora* species, DM pathogens are specialised for biotrophy, depending entirely on living plant tissue for growth and reproduction (Fabro et al. 2011; Asai et al. 2014). These pathogens use specialised intracellular structures called haustoria to mediate nutrient uptake and secrete arrays of effector proteins to modulate host immunity. To date, the studies on these pathogens have focused primarily on characterising their effectors, the evolutionary mechanisms underlying their success, and sustainable strategies to manage their impact on crops.

RNA interference (RNAi) has emerged as a promising tool for managing plant diseases. Host‐induced gene silencing (HIGS) and spray‐induced gene silencing (SIGS) are advanced RNAi‐based strategies that reduce pathogen virulence by targeting genes that are critical during infection (Bilir et al. 2022). HIGS involves the stable expression of double‐stranded RNA (dsRNA) constructs in transgenic plants, which silence pathogen genes through the transfer of small interfering RNAs (siRNAs) into the pathogen (Zand Karimi and Innes 2022). This approach has demonstrated efficacy in several systems; for example, HIGS in lettuce in *Bremia lactucae* significantly reduced infection by targeting the effector *HAM34* (Govindarajulu et al. 2015). Similarly, HIGS targeting core pathogenicity genes (e.g., *Dicer‐like*‐*DCL*‐ or RxLR effector genes) has shown promise in controlling *Plasmopara viticola* (Haile et al. 2021).

SIGS, a non‐transgenic RNAi‐based strategy, has shown promise for controlling plant pathogens by spraying dsRNAs directly onto crops (Amanda et al. 2023). This method induces gene silencing in pathogens by targeting essential genes in pathogens, reducing disease severity. SIGS offers significant advantages as an environmentally friendly alternative to chemical pesticides, particularly against eukaryotic plant pathogens like oomycetes. Effective application of SIGS requires careful design of dsRNA constructs, stability against environmental degradation and efficient uptake by the target pathogen.

Multiple studies have demonstrated the feasibility of using dsRNA for controlling oomycete pathogens. Ivanov and Guseva (2023) showed that exogenous dsRNA targeting *Phytophthora infestans* elicitin genes reduced the severity of lesions on potato explants. Similarly, Kalyandurg et al. (2021) demonstrated significant mitigation of disease symptoms by spraying potato leaves with dsRNA targeting key developmental and pathogenicity‐related genes in *P. infestans* ; this significantly mitigated disease symptoms. Sundaresha et al. (2022) also achieved enhanced disease resistance by targeting multiple *P. infestans* genes involved in infection and sporulation using in vitro synthesised dsRNAs.

Despite the successes, the broader adoption of SIGS faces challenges related to dsRNA delivery, stability and uptake by pathogens. Studies by Wang et al. (2023) and Zheng et al. (2024) addressed these issues by using advanced carriers such as functionalised carbon dots and biomimetic nanovesicles, which significantly improved dsRNA stability and uptake efficiency. The addition of nanoclay carriers (Sundaresha et al. 2022) enhanced pathogen resistance and duration of stability of dsRNAs under field conditions. Mechanistic investigations revealed variability in dsRNA uptake efficiency across *P. infestans* cell types and developmental stages, highlighting the need for improved delivery strategies (Qiao et al. 2021).

While most research has focused on controlled laboratory conditions, environmental factors influencing dsRNA efficacy remain underexplored. Studies like Hoang et al. (2022) emphasised the need for understanding the barriers to dsRNA uptake in real‐world settings, while Sundaresha et al. (2022) provided preliminary simulations of field applications with promising results. Taken together, these studies demonstrate the potential of SIGS as a viable strategy against oomycete pathogens. However, further research is required to optimise delivery systems, assess environmental persistence, and validate efficacy through extensive field trials to enable practical deployment of SIGS in agriculture.

In our previous studies, we targeted the *Hpa‐CesA3* gene using small RNAs (sRNAs) in sense and antisense forms and found that double‐stranded sRNAs were more effective at inhibiting pathogen infection than antisense sRNAs alone (Bilir et al. 2019). Here, we further investigated the use of short‐synthesised dsRNAs (SS‐dsRNAs) to target two genes across three DM pathogens, demonstrating their effectiveness for silencing essential structural genes and their broader applicability to other DM species. We also assessed the efficacy of SS‐dsRNAs in silencing dual and triple gene targets, confirming that multiple genes can be effectively suppressed in parallel. Additionally, we evaluated dsRNAs of varying lengths and identified limitations in dsRNA uptake by DM spores. Using confocal microscopy, we visualised fluorescent SS‐dsRNA internalisation in DM spores and germ tubes and compared the gene‐silencing effectiveness of chemically synthesised, in vitro‐transcribed, and *Escherichia coli* ‐expressed dsRNAs.

## 2 Results

### 2.1 Silencing *CesA3* Gene Across Different DM Pathogens Inhibits Germination and Infection

Previously, we focused on the *Arabidopsis thaliana–Hpa* interactions using *Hpa‐CesA3* in our sRNA experiments (Bilir et al. 2019). We took this further by investigating whether the same approach could be used in other obligate DM pathogens, such as *Peronospora viciae* f. sp. *pisi* (*Pvp*) and *Bremia lactucae* (*Bl*), DM pathogens of pea and lettuce, respectively. Using the *Hpa‐CesA3* sequence as the query, we conducted searches against genomic sequences of recently assembled *Pvp* (Webb et al., NIAB, Cambridge, UK, personal communication) and publicly available *Bl* genome sequences using BLASTN and BLASTX (Altschul et al. 1997). High‐confidence orthologs were identified based on significant sequence similarity and conserved domain architecture. The identified orthologs were designated as *Pvp‐CesA3* for pea‐infecting DM and *Bl‐CesA3* for lettuce‐infecting DM. *Hpa‐CesA3* was 83% to *Pvp‐CesA3* and 81% identical to *Bl‐CesA3*, while *Pvp‐CesA3* was 79% identical to *Bl‐CesA3* (Figure S1).

**FIGURE 1:**
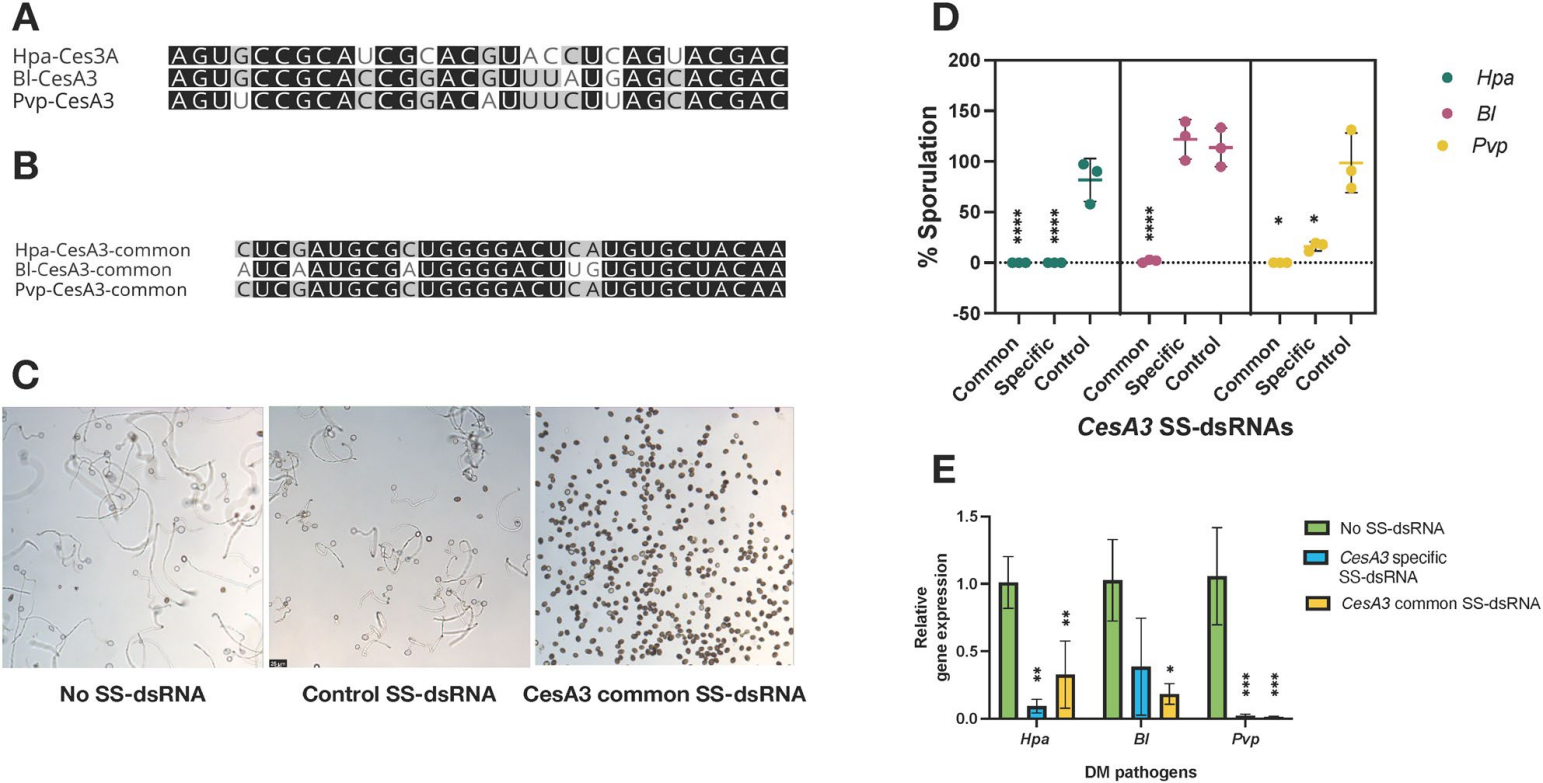
Targeting *CesA3* genes in *Hyaloperonospora arabidopsidis* (*Hpa), Bremia lactucae (Bl*) and *Peronospora viciae* f. sp. *pisi* (*Pvp*) with short synthesised double‐stranded RNAs (SS‐dsRNAs) inhibit spore germination and infection. (A) *Hpa‐CesA3*‐specific SS‐dsRNA targeting *Hpa‐CesA3* was designed based on sequences from Bilir et al. (2019), and alignment with *Bl‐CesA3* and *Pvp‐CesA3* sequences was performed. (B) *Hpa‐CesA3* common SS‐dsRNA targeting a conserved region of *CesA3* across *Hpa*, *Bl* and *Pvp* was designed using alignment data. (C) Spore germination assays were conducted using the designed SS‐dsRNAs. Representative images of *Pvp* spore germination are shown. (D) Sporulation assays were performed with the SS‐dsRNAs, assessing at 7 days post‐inoculation (dpi) for *Hpa* and *BI*, and at 8 dpi for *Pvp*, expressed as a percentage of the control. Control dsRNAS were designed from *HAC1Cala2* for *Hpa* and from *HpaG814274* for *Pvp* and *Bl*. (E) Expression levels of *CesA3* genes in all three pathogens were measured by reverse transcription‐quantitative PCR (RT‐qPCR) at 4 dpi. Statistical significance was analysed using a one‐way ANOVA followed by Dunnett's post hoc test, comparing the means of SS‐dsRNA‐treated and control samples, with correction for multiple comparisions under the assumption of normality. Values marked with an asterisk (*) indicate significant differences from the control group. Bars represent the standard deviation of three independent replicates. Adjusted *p*‐values for sporulation are as follows: *Hpa*: **** (*p* < 0.0001); *BI*: *CesA3* common **** (*p* < 0.0001); *Pvp*: *CesA3*‐common * (*p* = 0.0169), *Hpa*‐*CesA3*‐specific * (*p* = 0.0432). Adjusted *p*‐values for RT‐qPCR are as follows: *Hpa*: *Hpa*‐*CesA3‐* specific **(*p* = 0.0016), *CesA3*‐common **(*p* = 0.0070). *BI*: *CesA3*‐common *(*p* = 0.0167). *Pvp*: *Hpa*‐*CesA3*‐specific **(*p* = 0.0016), *CesA3*‐common **(*p* = 0.0015).

Previously, we targeted the *Hpa‐CesA3* gene using sense and antisense sRNAs individually and found that sRNA duplexes (dsRNAs) were more effective than antisense sRNAs alone (Bilir et al. 2019). However, in this current study, only 30 bp SS‐dsRNAs were used. We first checked the original *Hpa‐CesA3*‐specific 30 bp SS‐dsRNA against the *Bl‐CesA3* and *Pvp‐CesA3* genes, and the sequence alignment in this area showed 73.3%–76.6% identity, respectively (Figure 1A). Secondly, we also designed a new SS‐dsRNA, designated *CesA3*‐common, from a highly conserved region of the alignment (Figure S1) where the sequence identities were 100% for *Pvp* and 83.3% for *BI* (Figure 1B). Next, we checked for off‐target matches by subjecting the SS‐dsRNA sequences to BLAST searches against the genomic sequences of respective pathogens (*Hpa*, *Pvp* and *Bl*) and the host plant (*Arabidopsis*, pea and lettuce) genomes. The sequences did not show any detectable similarity to non‐target genes and host‐plant genomes.

To evaluate the effectiveness of the designed duplexes, we conducted in vitro germination and in planta sporulation assays with SS‐dsRNAs targeting the *CesA3* gene in three DM pathogens. For analysis of germination and sporulation inhibition, percentages were calculated relative to the control group. Spores from *Hpa*, *Pvp* and *Bl* in the untreated control (no SS‐dsRNA) or those treated with a control SS‐dsRNA (*HACCala2* for *Hpa* and *HpaG814274* for *Pvp* and *Bl*) germinated well. In contrast, treatment with the *Hpa‐CesA3*‐specific or the *CesA3*‐common SS‐dsRNAs significantly inhibited spore germination across all pathogens (Figure 1C shows a representative image). For the in planta assays, sporulation varied by pathogen. In *Hpa*, both the *CesA3*‐common and *Hpa‐CesA3‐*specific SS‐dsRNAs significantly inhibited sporulation (****p < 0.0001), while the negative control SS‐dsRNA (*HACCala2*) allowed full sporulation (Figure 1D) without any host response. Similarly, in *Bl*, the *CesA3*‐common SS‐dsRNA significantly reduced sporulation (****p < 0.0001), whereas the *Hpa‐CesA3*‐specific and control SS‐dsRNAs (*HpaG814274*) allowed full sporulation, highlighting the effects of single‐nucleotide mismatches between the SS‐dsRNAs and the pathogen target. In *Pvp*, the *CesA3*‐common SS‐dsRNA completely inhibited sporulation (*p = 0.0169), while the *Hpa‐CesA3*‐specific SS‐dsRNA significantly reduced sporulation (*p = 0.0432). The negative control SS‐dsRNA (*HpaG814274*) permitted full sporulation (Figure 1D).

We then assessed the expression levels of *CesA3* genes in infected tissues using reverse transcription‐quantitative PCR (RT‐qPCR) for each DM pathogen separately, following treatment with the *Hpa‐CesA3*‐specific and the *CesA3*‐common SS‐dsRNAs in all three DM pathogens. There were statistically significant reductions in the expression levels of *CesA3* genes in all tested pathogens (Figure 1E). In *Hpa*, both *Hpa‐CesA3*‐specific (**p = 0.0016) and *CesA3*‐common (**p = 0.0071) SS‐dsRNAs significantly reduced gene expression. Similarly, in *BI*, the *CesA3*‐common SS‐dsRNA (*p = 0.0167) led to a significant reduction. In *Pvp*, both *Hpa‐CesA3*‐specific (**p = 0.0016) and *CesA3*‐common (**p = 0.0015) SS‐dsRNAs also significantly reduced expression. These results correlated with the sporulation assays, confirming that SS‐dsRNA application effectively silences the targeted genes. These findings suggest that *CesA3* genes from different DM pathogens can be targeted using a single SS‐dsRNA. Additionally, SS‐dsRNAs can be designed either for specific pathogens or for a broader class of pathogens by targeting conserved gene regions.

### 2.2 Targeting *BTUB* Gene in *Hpa*, *Pvp* and *Bl* Further Confirms Gene Silencing in Downy Mildews

**FIGURE 2:**
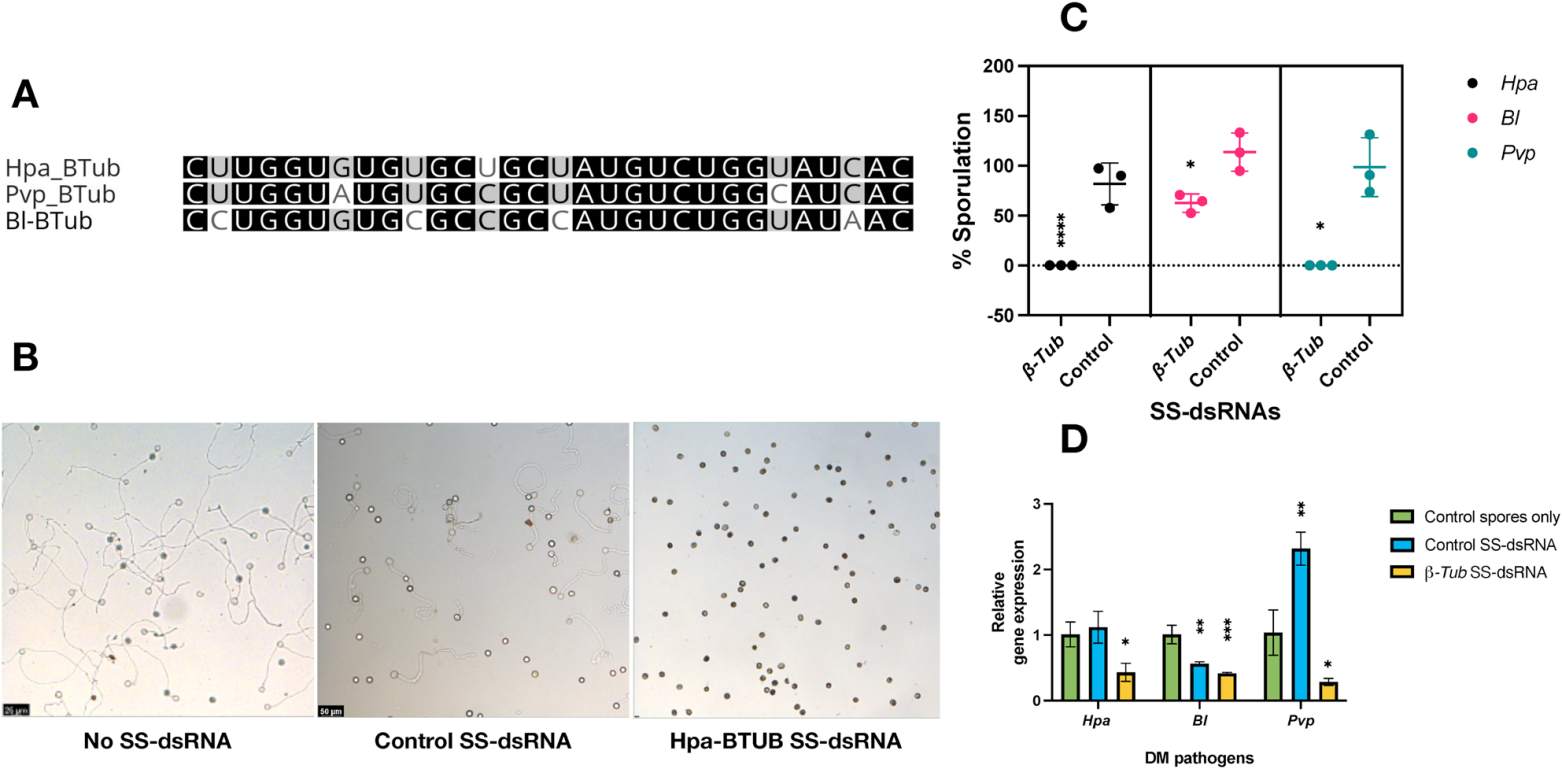
Silencing *BTUB* genes in *Hyaloperonospora arabidopsidis* (*Hpa), Bremia lactucae (Bl*) and *Peronospora viciae* f. sp. *pisi* (*Pvp*) inhibits spore germinations and alter sporulation. (A) A 30 bp short synthesised double‐stranded RNA (SS‐dsRNA) was designed form a conserved region identified through sequence alignment. (B) Spore germination assays were carried out using the designed SS‐dsRNAs. Representative images of *Hpa* spore germination are shown. (C) Sporulation assays were performed with the SS‐dsRNAs, assessing *Hpa*, *Bl* and *Pvp* sporulation at 7 days post‐inoculation (dpi), expressed as a percentage of the control. Control dsRNAS were designed from *HAC1Cala2* for *Hpa* and from *HpaG814274* for *Pvp* and *Bl.* (D) Expression levels of *BTUB* genes in all three pathogens were measured by reverse transcription‐quantitative PCR (RT‐qPCR) at 4 dpi. Statistical significance was assessed using a one‐way ANOVA followed by Dunnett's post hoc test, comparing the means of SS‐dsRNA‐treated and control, with correction for multiple comparisions under the assumption of normality. Values marked with an asterisk (*) indicate significant differences from the control group. Error bars represent the standard deviation of three independent replicates. Adjusted *p*‐values for sporulation: *Hpa*: **** (*p* < 0.0001); *BI*: * (*p* = 0.0273); *Pvp*: * (*p* = 0.0169). Adjusted *p*‐values for RT‐qPCR are as follows: *Hpa*: *(*p* = 0.0193), *BI*: ***(*p* = 0.0002), *Pvp*: *(*p* = 0.0180) and Control‐SS‐dsRNA (*HpaG814274*) **(*p* = 0.0011).

To confirm our results with *CesA3* and demonstrate that conserved genes can be used as targets in different DM pathogens, we applied the same approach to the *BTUB* gene in *Hpa*, *Pvp* and *Bl*. We used reciprocal BLASTN and BLASTX (Altschul et al. 1997) searches of *Hpa‐BTUB* against genomic sequences and identified its orthologs in *Hpa*, *Pvp* and *Bl*; the identified orthologs were designated as *Pvp‐BTUB* and *Bl‐BTUB*. Multiple sequence alignments (Figure S2) indicated that *Hpa‐BTUB* was 87% and 84% identical to *Pvp‐BTUB* and *Bl‐BTUB*, respectively, and that the amino acid sequences of these three genes contained the β‐tubulin domain IPR002453. Then, we designed a 30‐nt SS‐dsRNA from a highly conserved region of the alignment (Figure S2) where the sequence identities were 83.3% for *Bl* and 90% for *Pvp* (Figure 2A) and the BLAST searches against the pathogens' respective host genomes did not identify any detectable sequence similarity to non‐target genes.

In vitro spore germination assays showed that a single SS‐dsRNA that targets *BTUB* genes inhibited spore germination in all three DM pathogen spores (Figure 2B). In planta sporulation assays with SS‐dsRNA resulted in total inhibition of sporulation in both *Hpa*‐ (****p < 0.0001) and *Pvp*‐infected (*p = 0.0169) *Arabidopsis* and pea plants, respectively. Additionally, after treatment with SS‐dsRNA, *Bl* spores showed significantly reduced sporulation on lettuce plants (*p = 0.0273) (Figure 2C), indicating the effect of sequence variation within the SS‐dsRNA on silencing. Control‐SS‐dsRNAs (*HACCala2* for *Hpa* and *HpaG814274* for *Pvp* and *Bl*) allowed full sporulation of the pathogens.

Gene expression of *BTUB* in all three DM pathogens was quantified using RT‐qPCR after *Hpa‐BTUB* SS‐dsRNA treatment. A significant reduction in *BTUB* gene expression levels was observed across all tested pathogens (Figure 2D), with *Hpa* (*p = 0.0193), *Bl* (**p = 0.0002) and *Pvp* (*p = 0.0180). These results confirm that specific genes can be targeted across multiple DM‐causing species, and the observations with *BTUB* SS‐dsRNA align with those observed for *CesA3* SS‐dsRNA, demonstrating broader applicability rather than being limited to a single target.

### 2.3 The Lengths of SS‐dsRNAs Influence Their Uptake by Spores

Previously, we used 24‐, 25‐ and 30‐nt single‐stranded antisense sRNAs to investigate their effects on pathogen sporulation (Bilir et al. 2019). To advance this work and explore size constraints on dsRNA uptake by spores, we expanded our analysis to include a broader range of dsRNA lengths. Chemically synthesised *CesA3* SS‐dsRNAs (21–75 bp) were tested for their impact on spore germination rates in *Hpa* and *Pvp* (Table 1). Significant differences were observed in the effects of SS‐dsRNA lengths on spore germination for both pathogens.

**TABLE 1:** Effect of *CesA3* common short synthesised double‐stranded RNA (SS‐dsRNA) length on spore germination rates of *Hyaloperonospora arabidopsidis* (*Hpa*) and *Peronospora viciae* f. sp. *pisi* (*Pvp*).

Target Gene	SS‐dsRNA length (bp)	% *Hpa* spore germination^a^	% *Pvp* spore germination^a^
*CesA3*	21	154.72 (±13.66)	73.90 (±1.20)
	22	114.33 (±12.17)	73.05 (±1.25)
	23	115.66 (±11.45)	77.46 (±1.19)
	24	153.11 (±65.69)	75.64 (±0.99)
	25	247.10 (±45.88)	76.69 (±1.00)
	30	0	0
	50	0	0
	75	0	0

Abbreviation: bp, base pair.

^a^ Spore germination rates are expressed as percentages relative to unteated controls, calculated as (treatment value/control value) × 100. Values shown are means of three replicates ± standard deviation.

For *Hpa*, shorter dsRNAs (21–25 bp) exhibited variable spore germination rates, with the highest rate at 25 bp (247.10%) and the lowest at 22 bp (114.33%). In contrast, longer dsRNAs (30, 50 and 75 bp) completely inhibited spore germination. Similarly, for *Pvp*, dsRNAs in the 21–25 bp range resulted in germination rates ranging from 73.05% to 77.46%, with minimal variation among these lengths. As with *Hpa*, dsRNAs of 30, 50 and 75 bp entirely inhibited spore germination.

Because it was not feasible to synthesise dsRNA longer than 75 bp, we used in vitro‐transcribed (IVT) or *E. coli* ‐produced dsRNAs of various lengths. DNA fragments of *Hpa‐CesA3* (285 bp), *Hpa‐BTUB* (273 bp) and *E. coli β‐glucuronidase* (*GUS*) (255 bp, used as a negative control) were cloned and expressed in *E. coli* (Figure S3). Germination assays with *Hpa* spores showed that while *E. coli* ‐produced dsRNAs did not completely inhibit germination (Figure S4), they significantly reduced germination rates, with values of 79.52% for *Hpa‐CesA3*, 66.61% for *Hpa‐BTUB* and 48.65% for *GUS*, relative to untreated controls. Notably, when these dsRNAs were digested with RNase III and retested, all RNase III‐treated samples resulted in complete inhibition of spore germination. As no germinated spores were observed in any replicate, these results are reported qualitatively due to the absence of countable events.

Importantly, the *GUS* dsRNA, although lacking sequence similarity to the *Hpa* genome, still reduced germination, highlighting that not all dsRNAs are biologically inert. This underscores the need to empirically validate negative controls in each pathosystem. While *GUS* may be appropriate in other systems, our data demonstrate that dsRNA effects can be sequence‐independent and influenced by factors such as length and structure. Therefore, caution is required when selecting dsRNA sequences as negative controls in RNAi experiments.

**FIGURE 3:**
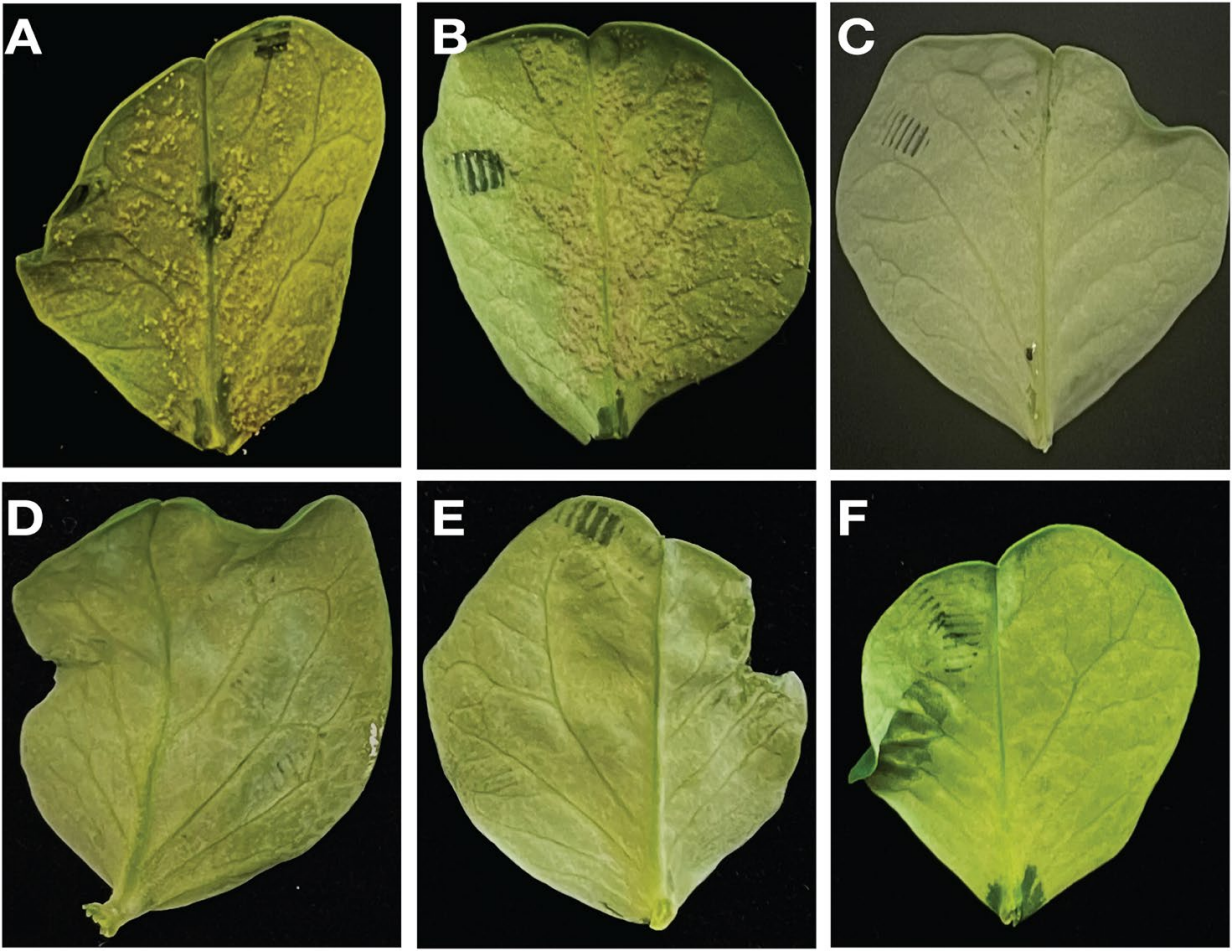
Effects of double‐stranded RNA (dsRNA) length on sporulation in pea leaves. Pea leaves were drop‐inoculated with *Peronospora viciae* f. sp. *pisi* (*Pvp*) spores mixed with *CesA3* dsRNAs of varying lengths, and sporulation was assessed at 7 days post‐inoculation. (A) Control leaf inoculated with spores only. (B) Control leaf inoculated with spores plus 100 bp non‐target dsRNA (designed from *HpaG803993*) showing normal sporulation. (C) Leaf inoculated with spores plus 30 bp SS‐*CesA3* common dsRNA. (D) Leaf inoculated with spores plus 50 bp SS‐*CesA3* dsRNA. (E) Leaf inoculated with spores plus 75 bp SS‐*CesA3* dsRNA. (F) Leaf inoculated with spores plus 100 bp in vitro‐transcribed *CesA3* dsRNA.

We next tested 50, 75 and 100 bp *CesA3* SS‐dsRNAs in sporulation assays using both *Hpa* and *Pvp*. In control pea plants infected with spores alone or with a 100 bp control dsRNA (designed from *HpaG803993*), normal sporulation was observed, as expected (Figure 3A,B). In contrast, the 30, 50 and 75 bp SS‐dsRNAs, as well as the 100 bp in vitro‐transcribed (IVT) *CesA3* dsRNA, completely inhibited pathogen sporulation (Figure 3C–E).

**FIGURE 4:**
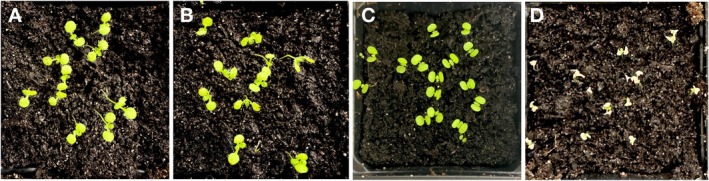
Impact of *Hpa‐CesA3* double‐stranded RNA (dsRNA) length on *Arabidopsis* responses. Seven‐day‐old *Arabidopsis* cotyledons were drop‐inoculated with *Hyaloperonospora arabidopsidis* (*Hpa*) spores mixed with either 30 or 75 bp *CesA3* dsRNAs and evaluated at 4 days post‐inoculation. (A) Control seedlings inoculated with spores only. (B) Seedlings treated with spores plus 30 bp control short synthesised (SS)‐dsRNA (designed from *HAC1Cala2*). (C) Seedlings treated with spores plus 30 bp SS*‐CesA3* common dsRNA. (D) Seedlings treated with spores plus 75 bp *Hpa‐CesA3* dsRNA.

Similar results were observed in the *Arabidopsis–Hpa* pathosystem. Control seedlings infected with spores alone (Figure 4A) and those treated with a 100 bp control dsRNA (designed from *HAC1Cala2*) (Figure 4B) showed normal sporulation. The 30 bp SS‐dsRNA did not induce any visible symptoms (Figure 4C); whereas the 75 bp SS‐dsRNA caused chlorosis on cotyledons and seedling necrosis by 4 days post‐inoculation (dpi) (Figure 4D).

Building on this, we used 100 bp IVT‐produced *CesA3* dsRNA and tested its effect on *Pvp* spore germination and infection. The 100 bp dsRNA completely inhibited spore germination. In sporulation assays with *Pvp*, the 100 bp dsRNA produced results similar to the 50 and 75 bp dsRNAs, with no sporulation observed.

### 2.4 The Concentration of SS‐dsRNAs Affects Both the Germination Rate and the Sporulation Rate of *Hpa*

We have established a high‐throughput screening protocol in *Hpa* to decipher gene functions using SS‐dsRNA. Our previous study (Bilir et al. 2019) demonstrated the dose‐dependent effects of dsRNAs on sporulation inhibition. Building on this, we targeted 11 genes (Table 2, Tables [Link], [Link] and [Link], [Link]) and optimised the concentration to use in our programme. Because dsRNAs were found to be more effective than antisense sRNAs, we tested lower concentrations (1 and 5 μM) for SS‐dsRNAs, compared to the higher concentrations (5, 10 and 20 μM) used for antisense sRNAs.

**TABLE 2:** Germination and sporulation rates of *Hyaloperonospora arabidopsidis* (*Hpa*) following gene targeting using two short synthesised double‐stranded RNA (SS‐dsRNA) concentrations.

Target gene IDs	% Germination^b^			% Sporulation^b^		
	SS‐dsRNA^a^ concentrations					Statistical significance^c^
	1 μM	5 μM	Statistical significance^c^	1 μM	5 μM	
*HpaG814170*	0	0	N/A^d^	152.24	0	****
*HpaG812660*	33.82^b^	0	*	87.76	0	****
*HpaG811651*	117.74	0	*	139.30	0	**
*HpaG800673*	183.44	0	**	75.90	33.76	*
*HpaG801549*	145.32	0	*	56.61	35.71	****
*HpaG809280*	70.74	0	**	106.97	50.79	**
*HpaG813100*	129.52	0	**	65.75	70.65	n.s
*HpaG810056*	283.43	244.39	n.s	199.50	22.02	***
*HpaG802074*	249.61	37.09	**	79.25	14.35	****
*HpaG802307*	117.19	184.11	*	33.43	50.87	*
*HpaG813395*	137.74	197.77	n.s	124.38	42.90	n.s

^a^ SS‐dsRNA: Short synthesised 30‐bp double‐stranded RNA.

^b^ Normalisation: Germination and sporulation rates are expressed as percentages relative to untreated *Hpa* controls, calculated as (treatment value/control value) × 100. Control values were set to 100% for each gene target.

^c^ Statistical significance (1 vs. 5 μM): Results of unpaired *t* test; significance levels: ns = not significant.

^d^ *HpaG814170* germination: Both 1 and 5 μM resulted in 0% germination (complete inhibition). Statistical comparison not applicable (N/A).

* *p* < 0.05.

** *p* < 0.01.

*** *p* < 0.001.

**** *p* < 0.0001.

When targeting gene *HpaG814170*, no germination was observed at either concentration. However, at 1 μM, sporulation increased statistically significantly to 152.24%, whereas no sporulation occurred at 5 μM. In the case of *HpaG812660*, germination was 33.82% at 1 μM, but both germination and sporulation were completely inhibited at 5 μM. Similarly, *HpaG811651* exhibited germination and sporulation rates of 117.74% and 139.30%, respectively, at 1 μM. However, at 5 μM, both processes were entirely suppressed. These findings suggest a concentration‐dependent regulatory effect of SS‐dsRNA on *Hpa* development, warranting further investigation into the underlying mechanisms. Conversely, some SS‐dsRNAs, such as those targeting *HpaG810056*, did not inhibit germination and sporulation; in fact, germination was enhanced at 1 μM (283.43%), though sporulation was reduced statistically significantly at 5 μM (22.02%).

Similarly, silencing *HpaG800673* and *HpaG801549* reduced sporulation to below 36% at 5 μM, but germination remained high at 1 μM, reaching 183.44% and 145.32%, respectively. SS‐dsRNAs targeting *HpaG802074* and *HpaG802307* exhibited moderate efficacy, with sporulation being more effectively inhibited at 5 μM, while germination showed either a slight reduction or enhancement.

For *HpaG813395*, the results were more variable, with germination rates increasing to 137.74% at 1 μM and 197.77% at 5 μM compared to the control, whereas sporulation decreased from 124.38% at 1 μM to 42.90% at 5 μM relative to the control (Table 2). Overall, these findings indicate that SS‐dsRNA efficacy depends not only on concentration but also on the target gene, as determined by the combined effects on spore germination and inhibition of sporulation. Some SS‐dsRNAs enhanced germination or sporulation at lower concentrations while effectively inhibiting them at higher doses. Targeting certain genes, such as *HpaG814170* and *HpaG812660*, resulted in consistent inhibition at both concentrations, whereas targeting others, such as *HpaG810056*, led to substantial variations between the two concentrations tested. In contrast, targeting *HpaG813395* displayed no significant changes in germination or sporulation across different SS‐dsRNA concentrations (Table 2).

These results highlight the complexity of gene silencing in *Hpa* and suggest that some genes are more amenable to effective targeting with SS‐dsRNAs than others. Additionally, these data also indicate (a) the need for optimised concentration and gene selection to achieve consistent pathogen control, and (b) germination assays carried out in vitro may not always correlate with that of in planta sporulation assays.

### 2.5 Multiplexed SS‐dsRNA Silencing Inhibits *Hpa* Germination and Sporulation

**FIGURE 5:**
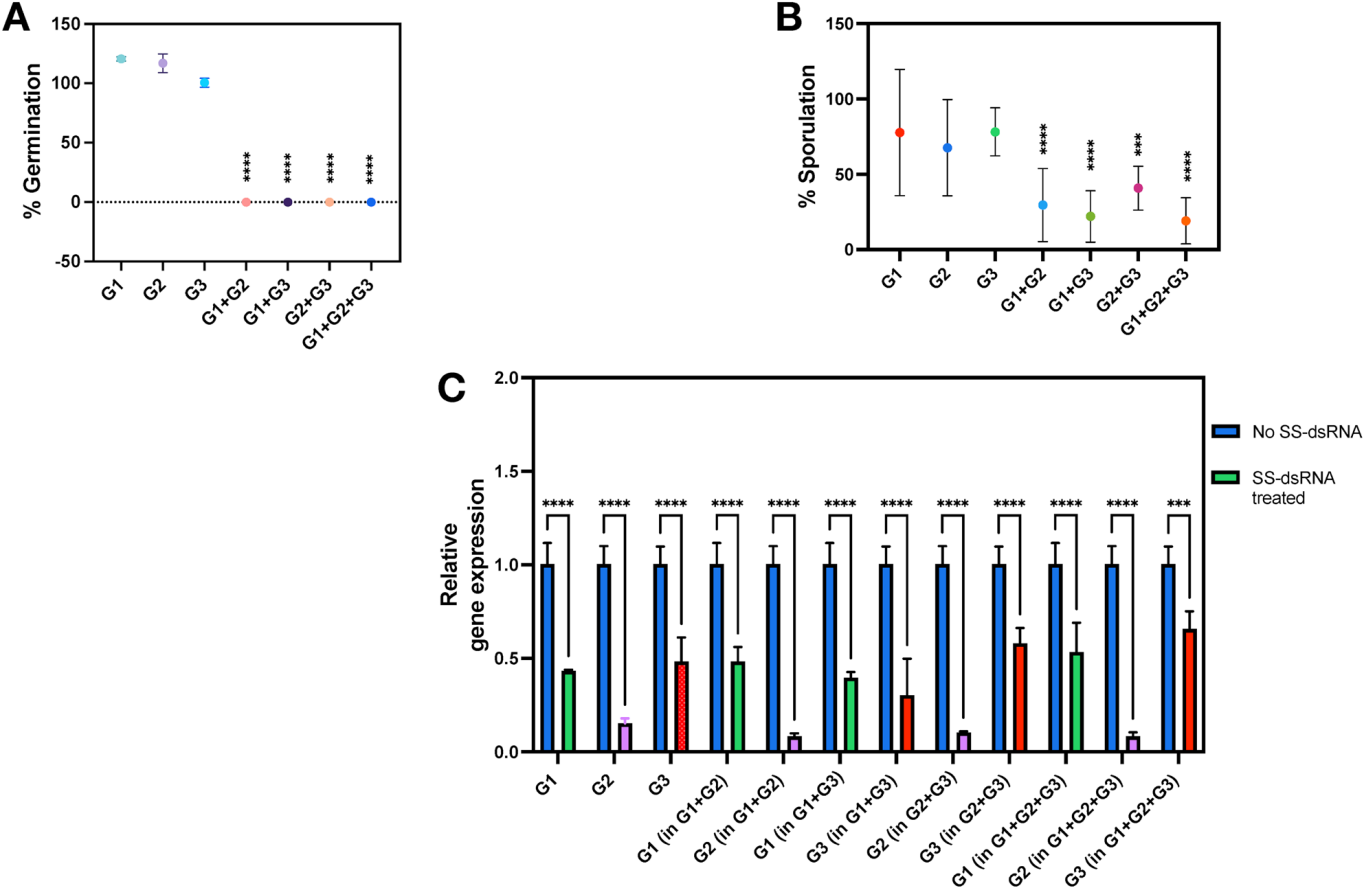
Effects of multiplexed short synthesised double‐stranded RNAs (SS‐dsRNAs) on the germination and sporulation of *Hyaloperonospora arabidopsidis* (*Hpa*). Three SS‐dsRNAs targeting different genes (*HpaG802064*, *HpaG802452*, and *HpaG803108*; G1, G2 and G3, respectively) were tested individually, as well as in pairs and triplets. (A) Germination rates assessed at 1 day post‐inoculation (dpi). (B) Sporulation assays were performed with the SS‐dsRNAs, assessing at 7 dpi for *Hpa*, expressed as a percentage of the control (no dsRNA). (C) Analysis of gene expression for each gene after SS‐dsRNA treatments using reverse transcription‐quantitative PCR. Gene expression levels were normalised to the control gene (*Actin*). Data are expressed using 2^−ΔΔ*C*t^ method to calculate the relative fold gene expression level of samples. For sporulation, statistical significance was assessed using a one‐way ANOVA followed by Dunnett's post hoc test, comparing the means of SS‐dsRNA‐treated and control, with correction for multiple comparisions under the assumption of normality. For gene expression analysis, two‐way ANOVA followed by Sidak's multiple comparisons test was used. Values marked with an asterisk (*) indicate significant differences from the control group. Bars represent the standard deviation of three independent samples. **** (*p* < 0.0001) and *** (*p* = 0.0009).

We wanted to investigate the efficacy of multiplex silencing using multiplexed SS‐dsRNAs. We selected three *Hpa* genes (*HpaG802064*, *HpaG802452* and *HpaG803108*, see Tables [Link], [Link] and [Link], [Link] for their possible functions; also referred to as *G1*, *G2* and *G3*, respectively in Figure 5), whose targeting did not cause full inhibition of spore germination and pathogen sporulation. The selection of target genes for the multiplexing study was based on the screening data in our research, similar to the approach used in the concentration effect study. In the spore germination assay, although individual SS‐dsRNAs did not cause any changes in spore germination rate (Figure 5A), targeting two or three genes simultaneously fully inhibited spore germination, demonstrating the additive effect of multiplexed silencing.

Similarly, in the sporulation assay, while individual SS‐dsRNAs caused only non‐significant reductions in sporulation rate, targeting two or three genes simultaneously enhanced suppression of sporulation (Figure 5B), confirming further the additive effect of multiplexing. To determine whether targeting multiple genes with dsRNA enhances suppression of pathogen sporulation (Figure 5B), we performed multiple pairwise comparisons among single and multiplexed dsRNA treatments. Sporulation was significantly reduced in *G1* + *G2* (*p = 0.0145), *G1* + *G3* (*p = 0.0049), and *G1* + *G2* + *G3* (**p = 0.0032) compared to *G1* alone. Comparable effects were observed for *G2* and *G3*, with particularly strong suppression when *G3* was included (e.g., *G3* vs. *G1* + *G3* and *G3* vs. *G1* + *G2* + *G3*; both ****p < 0.0001). These results indicate that adding additional targets enhances the suppression of sporulation, with the triple‐target construct showing the greatest reduction.

The gene expression data provided insight into SS‐dsRNA‐based silencing in the context of multiplexing. RT‐qPCR analysis confirmed that SS‐dsRNA treatments significantly reduced expression levels of the targeted genes (Figure 5C). All treatments—whether targeting single genes or in combination—resulted in strong and statistically significant knockdown (*** or ****, *p* < 0.001 or *p* < 0.0001, respectively). While multiplexed treatments targeting two or three genes did not always produce stronger suppression than single‐gene treatments, they consistently maintained effective knockdown across all targets. For example, the expression of *G3* (*HpaG803108*) was slightly lower in the single‐gene treatment than in the triple combination, highlighting some variability.

To assess whether multiplexing altered silencing efficiency of individual genes, we directly compared transcript levels of each gene in single‐target and multiplexed treatments. For *G1*, no significant differences in expression were observed between single and multiplexed treatments (*G1* vs. *G1* in *G1* + *G2*, *G1* + *G3*, and *G1* + *G2* + *G3*; all not significant). Similarly, *G3* expression was unaffected by co‐targeting in *G1* + *G3*, *G2* + *G3*, and *G1* + *G2* + *G3* combinations. In contrast, *G2* (*HpaG802452*) expression was significantly more suppressed in the multiplexed treatments *G1* + *G2* (**p = 0.004), *G2* + *G3* (*p* = *0.0249) and *G1* + *G2* + *G3* (**p = 0.004) compared to the *G2* single‐target treatment. These results suggest that multiplexing can enhance silencing efficiency for specific targets such as *G2*, without compromising specificity for others such as *G1* or *G3*.

These results support the conclusion that multiplexed SS‐dsRNA treatments are effective at reducing gene expression in *Hpa*, offering flexibility in targeting without loss of efficacy.

### 2.6 Confocal Microscopy Detects Uptake of Fluorescently Labelled SS‐dsRNA

We observed significant alterations in germination and sporulation rates upon targeting numerous genes with SS‐dsRNAs. To further explore these findings and provide mechanistic insight, we investigated whether spores of the DM‐causing pathogen actively internalise dsRNAs, thereby ruling out potential indirect effects. To investigate this, we employed confocal microscopy to visualise Cy5‐labelled SS‐dsRNAs in *Hpa* and *Pvp* spores. This approach allowed us to directly assess dsRNA uptake efficiency and variability in absorption among spores, strengthening the link between dsRNA exposure and the observed phenotypic effects. Pathogen spores were incubated with Cy5‐dsRNA for 6 h and imaged using a Nikon Ti2‐E AXE NSPARC laser point‐scanning confocal microscope. Fluorescent signals corresponding to Cy5‐labelled dsRNA were detected in or around spores and germ tubes.

**FIGURE 6:**
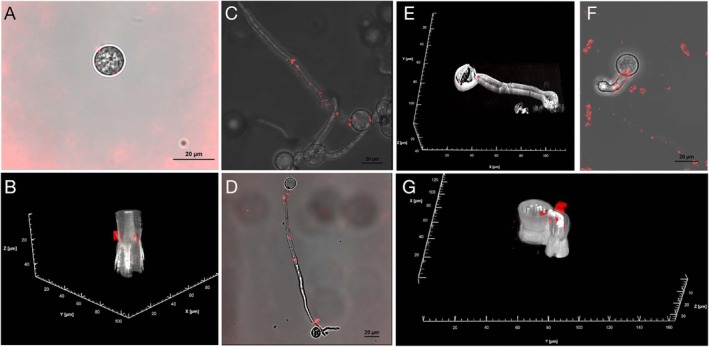
Internalisation of Cy‐5 labelled short synthesised double‐stranded RNAs (SS‐dsRNAs) by downy mildew spores. *Hyaloperonospora arabidopsidis* (*Hpa*) and *Peronospora viciae* f. sp. *pisi* (*Pvp*) were mixed with Cy‐5 labelled dsRNA and examined with a Nikon Ti2‐E AXE NSPARC laser point‐scanning confocal system 6 h after incubation. In all images, Cy‐5 labelled dsRNA is colour‐coded in red and superimposed over images of spores captured simultaneously with a transmitted detector mounted in the diascopic light path using 640 nm laser (grey colour‐coded). (A) *Hpa* spores, view from a single plane of a *Z*‐stack (77 slices@0.659 μm each step). (B) Same spores as A but 3D cropped rendered view. RNA particles (red coloured) inside spores. (C) Germinated *Pvp* spores view from a maximum intensity projection of a *Z*‐stack (61 slices@0.589 μm each step). (D) Germinated *Hpa* spores in a single plane view from a *Z*‐stack (77 slices@0.659 μm each step). (E) Germinated *Pvp* spores, view as a 3D cropped rendered projection of a *Z*‐stack (69 slices@0.589 μm each step). RNA particles imaged with NSPARC super resolution detector. (F) Germinated *Pvp* spores, view from a maximum intensity projection of a *Z*‐stack (61 slices@0.589 μm each step). (G) Same spore as F but in a 3D cropped rendered view of the *Z*‐stack. All images were captured with either a Nikon Plan Apochromat Lambda D 20× (0.8NA) or a Nikon Lambda S LWD water immersion 20× (0.95 NA) objective.

Single‐plane *Z*‐stack images (Figure 6A) and 3D‐rendered projections (Figure 6B) of *Hpa* spores showed discrete Cy5‐labelled RNA‐associated signals within or adjacent to spore structures. Similarly, in *Pvp* spores, Cy5‐labelled dsRNA signals were observed associated with germinated spores and germ tubes (Figure 6C–G). These microscopy observations are consistent with the association of exogenous dsRNA with spores of DM‐causing pathogens and support the functional evidence for dsRNA entry obtained from gene‐silencing experiments.

### 2.7 SS‐dsRNA‐Mediated Gene Silencing Is Sustained Over Time

To assess the efficacy and longevity of SS‐dsRNA‐mediated gene silencing, we targeted *HpaG809066*, a 1575 bp gene encoding a 525‐amino‐acid protein. Analysis using InterProScan (Blum et al. 2024) and UniProt (The UniProt Consortium 2025) revealed that this protein contains a signal peptide and a protein disulphide‐isomerase domain, suggesting a potential role in pathogen virulence or host interaction. A 30 bp SS‐dsRNA was designed to target *HpaG809066*. *Arabidopsis* leaves were drop inoculated with spores mixed with 5 μM SS‐dsRNA. Gene expression level was assessed using RT‐qPCR at 4, 7, 10 and 11 dpi.

**FIGURE 7:**
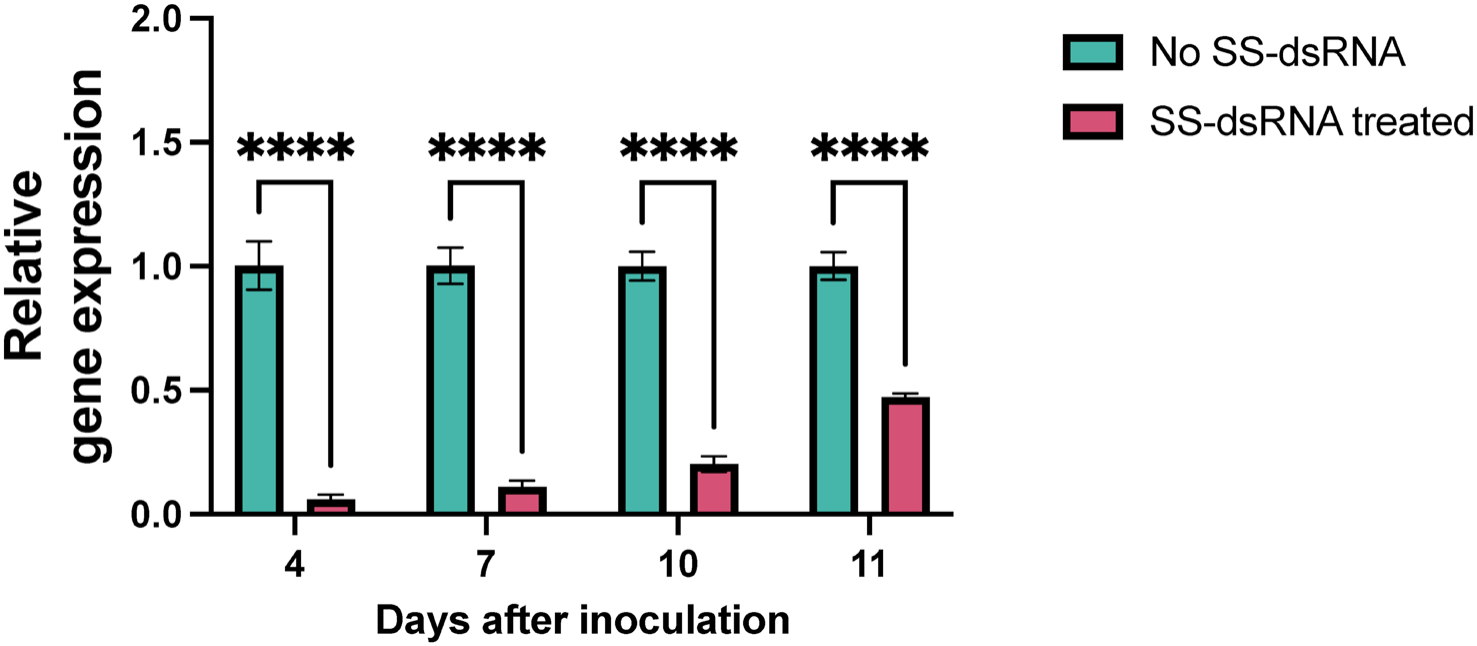
Short synthesised double‐stranded RNA (SS‐dsRNA)‐mediated silencing of *HpaG809066* reduces gene expression over time. *Hyaloperonospora arabidopsidis* (*Hpa*) spores were treated with 5 μM SS‐dsRNA, while untreated samples served as controls. Gene expression levels were assessed by reverse transcription‐quantitative PCR at 4, 7, 10 and 11 days after inoculation. A significant reduction in gene expression was observed in SS‐dsRNA‐treated samples compared to untreated controls at all time points (***, *p* < 0.0001). Statistical significance was assessed using a two‐way ANOVA followed by Sidak's multiple comparisons test, comparing the means of SS‐dsRNA‐treated and untreated samples. Values marked with an asterisk (*) indicate significant differences. Bars represent the standard deviation of three independent samples.

As shown in Figure 7, the level of gene expression remained high in untreated samples (no SS‐dsRNA). In contrast, SS‐dsRNA‐treated samples exhibited a significant and sustained reduction in expression: 94% at 4 dpi, 89% at 7 dpi, 80% at 10 dpi, and 53% at 11 dpi. The observed reduction was statistically significant (****p < 0.0001), indicating robust and lasting silencing induced by SS‐dsRNA.

## 3 Discussion

We used three host–DM pathosystems—*Arabidopsis–Hpa*, pea*–Pvp* and lettuce*–Bl*—to investigate factors influencing dsRNA‐mediated pathogen control. Through in vitro spore germination assays, in vivo sporulation assays and gene expression analyses, we demonstrated that exogenously applied SS‐dsRNAs targeting the *CesA3* and *BTUB* genes effectively reduced gene expression, thereby inhibiting spore germination and plant infection. The uptake efficiency of dsRNAs by spores was influenced by dsRNA length, while dsRNA concentration significantly affected spore germination and infection rates. Additionally, we demonstrated the feasibility of simultaneously targeting multiple genes using SS‐dsRNAs. Using confocal microscopy, we observed patterns consistent with the uptake of fluorescently labelled SS‐dsRNAs by *Hpa* and *Pvp* spores, supporting the functional evidence from gene‐silencing assays. Additionally, we showed gene silencing could be sustained for at least 11 days within *Hpa*. These findings highlight the potential of SIGS as an effective strategy against DM pathogens.

The oomycete cell wall is primarily composed of β‐glucans and cellulose (Raaymakers and Van den Ackerveken 2016), with cellulose biosynthesis mediated by cellulose synthase (CesA) enzyme complexes (Grenville‐Briggs et al. 2008). The CesA3 subunit, essential for cellulose biosynthesis, is a key target for oomycete‐targeting compounds such as carboxylic acid amides (CAAs), which disrupt cell wall formation in oomycetes (Blum et al. 2012). CAAs exhibit activity against oomycetes, including *P. infestans* , *Phytophthora capsici* and *Plasmopara viticola* (Blum and Gisi 2012), although specific amino acid changes in CesA3 can confer insensitivity (Blum et al. 2012).

Microtubules, composed of α‐ and β‐tubulin subunits, are crucial cytoskeletal components involved in cell division and plant–microbe interactions (Hardham 2013) (Piquerez 2011). β‐tubulin has been targeted in fungicides such as methyl benzimidazole carbamates (MBCs), which inhibit tubulin polymerisation but are ineffective against oomycetes (Vela‐Corcia et al. 2018). However, β‐tubulin inhibitor zoxamide is effective against oomycete pathogens, including *Phytophthora* and *Pythium* spp. (Cai et al. 2016).

Previous studies have demonstrated the role of *CesA3* and *β‐tubulin* genes in oomycete pathogenicity through mutant analyses and fungicidal treatments. However, our results demonstrate that these genes can also be effectively targeted using dsRNA‐based biopesticides. Until now, many pathogen genes have been targeted using SIGS‐based methods (Bilir et al. 2022). Similarly, dsRNA shows promise for pest management by silencing target genes. For example, dsRNAs targeting tubulin genes (*Msalpha‐tubulin* and *Msbeta‐tubulin*) in *Mythimna separata*, the oriental armyworm, effectively reduced target gene mRNA levels, leading to reduced body weight and increased mortality phenotypes (Wang et al. 2018). In our study, silencing of *BTUB* genes in three DM‐causing pathogens not only supports tubulin as a promising candidate for SIGS but also highlights that pathogen‐specific regions within these genes can enhance the specificity of dsRNA targeting. A unique aspect of our study is the use of 30 bp SS‐dsRNAs to target both the *CesA3* and *BTUB* genes in *Hpa*, *Pvp* and *Bl*, emphasising the importance of allelic variations within the targeted regions. For instance, while sporulation results for *Hpa* and *Pvp* were similar, those for *Bl* differed, likely due to the dsRNA mismatches in the target region.

The term “RNAi” describes the overarching mechanism of gene silencing mediated by dsRNAs through the association of sRNAs, such as siRNAs and miRNAs, with Argonaute effector proteins (Chou et al. 2024). Initially, RNAi was considered to be a sequence‐specific mechanism requiring perfect complementarity between the siRNA guide strand and the target mRNA sequence (Elbashir et al. 2001). However, subsequent studies revealed that the specificity of siRNA‐mediated silencing depends on both the position and identity of nucleotide mismatches (Qiu et al. 2005). For instance, certain mismatches, such as A:C pairs and G:U wobble base pairs, are well tolerated, enabling silencing efficiency comparable to fully matched sequences (Du et al. 2005). This tolerance to mismatches might explain the observed effects in our spore germination and sporulation assays, where mismatches at the target region could have been accommodated.

Recent studies have highlighted the importance of dsRNA length and concentration in determining the efficacy of RNAi‐mediated silencing (Höfle et al. 2019; Baldwin et al. 2018a; Koch et al. 2019; He et al. 2020; Soumya et al. 2024). Studies with longer dsRNAs (400–1500 bp) on *Fusarium graminearum* have shown greater effectiveness due to their ability to generate a more diverse pool of siRNAs (Höfle et al. 2019). However, researchers working on *Sclerotinia sclerotiorum* have argued that longer constructs (> 2 kb) are prone to degradation, reducing their applicability under field conditions (Soumya et al. 2024). In the present study, we used different lengths of chemically synthesised, IVT‐ or *E. coli‐*produced dsRNAs of different lengths and determined their impacts on *Hpa* and *Pvp* spore germination and sporulation on their host plants. While 21–25 bp did not inhibit spore germination entirely, 30, 50, 75 and 100 bp long dsRNAs effectively inhibited spore germination, indicating that spores could take up dsRNAs as long as 100 bp. When we pushed beyond the limits of chemically synthesising to longer fragments using *E. coli*‐produced dsRNA fragments *Hpa‐CesA3* (285 bp) and *Hpa‐BTUB* (273 bp), we did not see total inhibition of spore germination. However, digesting these long fragments with RNase III allowed full inhibition of spore germination. This clearly indicates that DM‐causing pathogen spores are limited in their uptake of dsRNA in respect of its length.

Åsman et al. (2016) used co‐immunoprecipitation to isolate sRNAs bound to AGO proteins in the oomycete pathogen *P. infestans* , finding a strong enrichment of 24–26 nucleotide sRNAs. Similarly, Jia et al. (2017) analysed sRNAs in *Phytophthora parasitica* and observed that 25–26 nucleotide sRNAs are linked to effective gene silencing in this species. Because only 30 bp SS‐dsRNAs consistently inhibited spore germination and sporulation, we used this length in all subsequent assays to ensure reliable gene silencing. Although the mechanism is not fully understood, it is possible that dsRNAs shorter than 30 bp either do not bind the target transcript effectively or fail to recruit the RISC complex for degradation. We also note the importance of properly choosing control dsRNA, as they can show off‐target effects. For example, when we used *E. coli* ‐produced dsRNA from *E. coli* *GUS* gene (255 bp) as a negative control, we observed reduced germination; after digestion with RNase III, we observed full inhibition of spore germination.

Efficient dsRNA uptake occurs in the fungal plant pathogens including *Botrytis cinerea*, *S. sclerotiorum* and *Verticillium dahliae*, but no uptake in the fungal pathogen *Colletotrichum gloeosporioides* and weak uptake in the fungus *Trichoderma virens* (Qiao et al. 2021). Interestingly, with the oomycete plant pathogen *P. infestans* , RNA uptake was limited and varied across different cell types and developmental stages (Qiao et al. 2021). Similarly, studies on the wheat–*Zymoseptoria tritici* pathosystem revealed that *Z. tritici* is incapable of dsRNA uptake (Kettles et al. 2019). In some cases, comparisons of dsRNA fragments targeting different genes have shown that shorter dsRNAs (~250–350 bp) are less effective in generating diverse siRNAs but can achieve gene‐specific effects under certain conditions (Baldwin et al. 2018b). In addition, these uptake efficiencies are linked to the functionality of fungal RNAi pathways, including *Dicer* and *Argonaute* genes, which have been shown to control RNAi effectiveness (Gaffar et al. 2019; Werner et al. 2019). However, longer dsRNAs are not always preferable, as they may increase the likelihood of off‐target effects in the host plant. This was evident in our experiments, where longer dsRNAs targeting *CesA3* induced chlorosis and necrosis in *Arabidopsis*. These symptoms were absent with shorter dsRNAs, suggesting non‐specific effects likely due to partial sequence homology with plant transcripts. To minimise such risks in future applications, dsRNA design should prioritise sequence specificity by avoiding regions with homology to host plant genes, verified through BLAST or siRNA off‐target prediction tools. Incorporating genome‐wide off‐target screening during design, using host transcriptome data, and functional validation of dsRNAs in non‐target plants can further reduce unintended effects. Thus, dsRNA design must weigh efficacy, stability and specificity to ensure both pathogen suppression and host safety.

The concentration of applied dsRNA is crucial and the optimum varies between pathosystems. Moderate concentrations (~30–100 ng/μL) typically achieve optimal silencing, whereas higher concentrations (> 200 ng/μL) may induce non‐specific stress responses or degradation (Qiao et al. 2021). Conversely, concentrations below 10 ng/μL are generally ineffective for pathogen gene silencing (Qiao et al. 2021; Koch et al. 2016). In our studies, we tested 10, 100 and 200 ng/μL (equivalent to 1, 5 and 10 μM) on *Hpa* and *Pvp* spore germination and infection. We found 100 ng/μL to be optimal for *Hpa* and *Pvp* spore germination assays. However, for sporulation, 100 ng/μL was optimal for the *Arabidopsis–Hpa* pathosystem, whereas 200 ng/μL was required for the pea–*Pvp* pathosystem. These findings highlight the necessity of optimising dsRNA concentration for each pathosystem.

Recent studies highlight the efficacy of dsRNA constructs that target multiple genes in fungal pathogens. For example, simultaneous silencing of multiple virulence and growth‐related genes in *F. graminearum* (including *FgSGE1*, *FgSTE12* and *FgPP1*) enhanced wheat resistance to Fusarium head blight (Wang et al. 2020) (Yang et al. 2021). Similarly, targeting ergosterol biosynthesis genes in *B. cinerea* effectively suppressed fungal proliferation (Danielle et al. 2021). In our multiplexing experiments, targeting specific individual genes did not inhibit spore germination. However, simultaneous silencing of two or three genes resulted in complete germination inhibition. Likewise, sporulation assays showed a significant reduction when multiple genes were targeted together. These findings reinforce the power of multiplex approaches in disrupting complex virulence networks, highlighting their potential for pathogen control across diverse pathosystems.

Several studies have demonstrated the uptake of fluorescently labelled dsRNAs in fungal and oomycete pathogens using confocal microscopy, confirming its localisation within targeted cells. For example, *Cercospora zeina* spores were shown to internalise fluorescein‐labelled dsRNA, leading to RNAi‐mediated silencing of GFP expression and fungal viability reduction (Marais et al. 2024). Similarly, fluorescein‐labelled dsRNA targeting the virulence gene *Tup1* in *F. oxysporum* confirmed dsRNA internalisation, which correlated with reduced gene expression and fungal pathogenicity (Fan et al. 2024). Other studies, such as those on *P. infestans* , used confocal microscopy to visualise dsRNA uptake in sporangia, linking RNA uptake to suppression of critical genes involved in infection (Kalyandurg et al. 2021). Remarkably, the emerging use of nanosheets or other delivery enhancements has improved dsRNA uptake efficiency and stability under diverse environmental conditions, further broadening the applicability of such approaches (Chen et al. 2022). Structural barriers, such as the integrity of spore walls, are likely contributors to this variability, prompting researchers to explore additional delivery strategies, such as enzymatic pretreatments or improved carriers (Qiao et al. 2021). Additionally, confocal microscopy studies have highlighted the importance of distinguishing dsRNA entry from surface adhesion, with methods like *Z*‐stack imaging and colocalization analysis providing evidence for intracellular localisation (Qiao et al. 2021). By combining fluorescence imaging techniques with functional RNAi assays, these studies have established strong correlations between dsRNA uptake, target gene silencing and pathogen inhibition. For example, the fluorescence intensity of dsRNA within fungal cells has been linked to reduced expression levels of key target genes, phenotypic outcomes such as growth retardation, and suppression of plant disease symptoms (Marais et al. 2024; Fan et al. 2024; Danielle et al. 2021).

In our study, we used Cy‐5 labelled SS‐dsRNAs and checked their internalisation by *Hpa* and *Pvp* spores. Confocal microscopy clearly indicates that these fluorescently labelled dsRNAs can be detected in or around spores or germ tubes, reinforcing the utility of confocal microscopy in validating RNAi delivery systems and contributing to a growing understanding of dsRNA internalisation mechanisms in oomycetes.

The observed discrepancy between the inhibition of pathogen germination on glass slides and the persistence of sporulation on the host plant with some of the SS‐dsRNA (Table 2) may arise from fundamental differences in the experimental environments and pathogen biology. In vitro germination assays provide a controlled system where dsRNA directly interacts with the pathogen under optimised conditions, potentially achieving higher effective concentrations and uninterrupted activity. In contrast, in planta assays introduce dynamic variables: host tissues may absorb or degrade applied dsRNA, reducing its bioavailability to the pathogen (Qiao et al. 2023). Additionally, the pathogen's interaction with the plant cuticle, apoplastic enzymes, or host‐derived nutrients could alter its physiological state, enabling partial evasion of dsRNA‐mediated silencing (Islam et al. 2021). The pathogen might also employ alternative genetic pathways during later infection stages, such as sporulation, that are less reliant on the genes targeted during germination. Furthermore, microenvironmental factors (e.g., humidity, leaf microbiota, or plant immune responses) could stabilise the pathogen or diminish dsRNA efficacy (Ray et al. 2022). These findings indicate the necessity of optimising dsRNA delivery mechanisms to enhance stability and uptake in planta, while highlighting the complementary value of both assays in elucidating gene function across distinct phases of the pathogen's life cycle.

SS‐dsRNA‐mediated gene silencing in *Hpa* exhibited a sustained reduction in gene expression over an 11‐day period, with the strongest suppression observed at 4 dpi (94% reduction) and a gradual decline to 53% by 11 dpi. This longevity aligns with previous studies indicating that RNA silencing duration depends on RNA stabilisation strategies and pathogen‐specific RNAi efficiency. Unlike naked dsRNA, which is prone to rapid degradation (Whisson et al. 2005), our SS‐dsRNA exhibited prolonged activity, suggesting enhanced stability and uptake by *Hpa*. While fungal pathogens such as *S. sclerotiorum and B. cinerea* generally exhibit more efficient dsRNA uptake and prolonged silencing, the oomycete pathogen *P. infestans* shows weaker RNA uptake, potentially explaining the decline in silencing at later time points (Han et al. 2025; Qiao et al. 2021; Danielle et al. 2021). Additionally, gene‐specific factors, such as the target sequence and its role in pathogen virulence, likely influenced silencing efficiency. The significant suppression observed here highlights SS‐dsRNA as a promising tool for managing DM, though further studies optimising dsRNA stabilisation and delivery could enhance its durability and field applicability.

In conclusion, our study demonstrates that exogenously applied SS‐dsRNAs effectively silence targeted genes in DM pathogens, inhibiting spore germination and reducing plant infection. The uptake efficiency of dsRNA varies with length and concentration, highlighting key parameters for optimising SIGS‐based pathogen control. While *CesA3* and *BTUB* genes are effective targets, pathogen‐specific sequence variations influence dsRNA specificity and efficacy. Our findings also reveal limitations in dsRNA uptake by oomycete spores, underscoring the need for precise sequence design to balance efficacy and minimise off‐target effects. Looking ahead, translating these findings to field applications will require addressing environmental stability, formulation and delivery challenges. Encapsulation of dsRNAs using biodegradable nanocarriers or clay‐based systems may enhance uptake, persistence and protection from degradation under field conditions. Integration with foliar sprays or seed treatments, combined with pathogen surveillance to inform target design, could enable scalable and crop‐specific RNA‐based biopesticides. These insights advance the foundation for sustainable, precision‐guided disease control strategies in agriculture.

## 4 Experimental Procedures

### 4.1 Plant Lines

*Arabidopsis* mutant Ws‐*eds1* (Parker et al. 1996) was used to maintain and test SS‐dsRNAs with *Hpa* Emoy2 isolate as described (Bilir et al. 2019). Marrowfat pea seeds were obtained from Church of Bures, https://churchofbures.co.uk, and used for pea‐DM experiments. For lettuce DM experiments, lettuce cv. Gustav's Salad was used, and seeds were purchased from a local garden centre.

### 4.2 Downy Mildew Isolates and Propagation

#### 4.2.1 *Hpa* Propagation

*Hpa* isolate *Emoy2* was maintained on *Arabidopsis* Ws‐*eds1* plants. The 7‐day‐old infected seedlings were collected in cold sterile distilled water (SDW), gently vortexed and filtered through a layer of Miracloth. The *Hpa* conidiospores were collected by centrifugation, washed twice with cold SDW, and the conidiospore concentration released in SDW was adjusted to 5 × 10^4^ spores/mL using a haemocytometer. The 7‐day‐old *Arabidopsis* plants were inoculated with the obtained *Hpa* conidiospores by using a spray atomiser. The inoculated plants were then placed in a plastic tray covered with transparent lids and the edges sealed with tape to maintain humidity. The plants were grown at 16°C under a 12‐h photoperiod in growth cabinets with a light intensity of 150 μmol m^−2^ s^−1^. Sporulation was assessed 7 dpi as described (Bilir et al. 2019).

#### 4.2.2 *Bl* Propagation

*Bl‐*Tid1 isolate was obtained from a natural infection on lettuce cv. Gustav's Salad, grown at an allotment in Tiddington, Stratford upon Avon. The inoculum was washed off, single spored, and subsequently maintained on the same cultivar. The inoculum was prepared in the same way as *Hpa*, adjusted to a concentration of 5 × 10^4^ spores/mL. The 7‐day‐old lettuce cotyledons were drop inoculated with 5–10 μL of inoculum onto each cotyledon. The inoculated plants were placed in a plastic tray, covered as described in Hpa and grown under the conditions specified in *Hpa*. Sporulation was assessed 7 dpi.

#### 4.2.3 *Pvp* Propagation

*Pvp* isolate DM3 was from the DM collection of NIAB, UK. The infected pea plants were collected in cold SDW, gently vortexed, and filtered through a layer of Miracloth. The spore suspension adjusted to 5 × 10^4^ spores/mL was used to inoculate 4‐day‐old pregerminated pea seeds. The seedlings were immersed in the spore suspension for 30 min with gentle shaking every 5 min to ensure uniform inoculation and then immediately sown in a compost and grown at 16°C under a 12‐h photoperiod in a growth cabinet. After 10 days, the inoculated plants were covered with a transparent lid for 2 days to aid the pathogen in sporulating.

### 4.3 Generation of Small dsRNAs

Small dsRNAs were designed using InvivoGen's, https://www.invivogen.com/sirnawizard/design_advanced.php, siRNA wizard software or home developed MYCIsiRNA https://mycosirna.wp.worc.ac.uk/design.html software by adjusting the motif size to 30 nt, and small dsRNA duplexes were either obtained as chemically synthesised ribonucleotides from Merck or GenScript. In addition, dsRNAs were also produced by IVT in‐house by using the MEGAscript kit (Thermo Fisher) according to the manufacturer instructions or obtained from RNA Greentech LLC. Sequences of dsRNA duplexes used are given in the Table [Link], [Link].

### 4.4 Production of dsRNAs in *E. coli*

Production and purification of dsRNA were carried out according to (Ahn et al. 2019) with some modification. Synthetic DNA fragments (G‐blocks) encoding partial sequences (273, 285 and 255 bp) of the *Hpa‐CesA3* and *Hpa‐BTUB* genes, and *E. coli* *GUS* gene as control, respectively, were ordered from Integrated DNA Technologies with added SacI and PstI restriction sites. Amplification of all G‐blocks was done using Taq DNA polymerase (New England Biolabs) along with gene‐specific primers. The PCR‐amplified *Hpa‐CesA3*, *Hpa‐BTUB and GUS* DNA fragments and pL4440 plasmid vector with two inverted T7 promoters were digested with SacI and PstI. Digested products were then ligated together at 4°C overnight in the presence of T4 DNA ligase. The constructs were then transformed into *E. coli* HT115 (DE3), which lacks RNase III, using a standard transformation protocol. Positive transformants were selected at 100 μg/mL ampicillin and 12.5 μg/mL tetracycline on Luria Bertani (LB) agar plates and verified by colony PCR. Single transformants of HT115 carrying the recombinant pL4440 vectors were inoculated into 4 mL of LB medium containing 100 μg/mL ampicillin and 12.5 μg/mL tetracycline, then incubated overnight at 37°C with shaking (190 rpm). A 500 μL aliquot of the overnight culture was transferred into 100 mL of LB broth containing the same antibiotics and incubated at 37°C with shaking to an OD_600_ of approximately 0.4. RNA transcription was induced by adding 1 mM isopropyl β‐D‐1‐thiogalactopyranoside (IPTG) (Thermo Fisher Scientific) and incubation continued for a further 5 h under the same conditions. The cultures were harvested for dsRNA extraction.

Total RNA was purified from 100 mL of a bacterial culture using the TRIzol Max Bacterial RNA Isolation Kit according to the manufacturer's instructions (Thermo Fisher). The RNA pellet was dissolved in nuclease‐free water and further treated with Turbo DNase and RNase A to remove DNA contamination and single‐stranded RNA contaminants. The dsRNA was further purified by adding chloroform/isoamyl alcohol 24:1, vol/vol (Sigma‐Aldrich). The upper aqueous phase containing the dsRNA was transferred into a new tube, mixed with 7.5 M ammonium acetate and isopropanol, and centrifuged at 12,000 *g* for 30 min at 4°C. The dsRNA pellet was washed twice with 70% ethanol, air‐dried, and resuspended in nuclease‐free water. RNA yield and quality were measured using a Synergy H1 hybrid reader (BioTek), while the RNA integrity was assessed by 1.2% agarose gel electrophoresis.

### 4.5 Germination Assays

*Hpa*, *Pvp* and *Bl* spores adjusted to 5 × 10^4^ spores/mL in SDW were mixed with small dsRNAs at a final concentration of 1 or 5 μM in an Eppendorf tube, then incubated on ice for 20 min. A total of 100 μL spore suspension mixed with or without dsRNA (control) was dropped onto the microscope glass slides with three biological replicates. Microscope glass slides were placed into the Petri dishes, which were covered with cling film to increase the humidity and placed into a plastic box. These boxes were then incubated with a 12 h light/12 h dark regime at 16°C for 24 h. Spores were examined under a light microscope (Leica DM5500 B and Olympus CK‐2) 24 h after incubation, and total and germinated spores were counted. The percent germination was calculated based on the control group representing only pathogen spores without dsRNA. The values were expressed as a percentage of control to eliminate variation encountered in the controls. Each experiment was repeated at least twice.

### 4.6 Sporulation Assays

A total of 100 μL of *Hpa*, *Bl* and *Pvp* spore suspensions, with or without short dsRNA, was prepared following the same procedure as in the germination assay. The only exception was for *Pvp*, where a final dsRNA concentration of 10 μM was used.

Seven‐day‐old *Arabidopsis* seedlings (for *Hpa*) and lettuce seedlings (for *Bl*), grown in plastic modules with uniform spacing, were used for inoculation. Three replicates were prepared for each pathogen, with 10–15 seedlings per replicate for *Hpa* and 3 seedlings per replicate for *Bl*. These seedlings were drop‐inoculated with the spore suspension, applying 1 μL to each cotyledon for *Hpa* and 10 μL to each cotyledon for *Bl*.

For *Pvp*, 4‐day‐old pregerminated pea seeds were sown in compost, kept under the same conditions as during propagation. After 5 days, the pea seedlings were inoculated by placing 10 μL of spore suspension inside the newly expanded leaves. All plants (*Arabidopsis*, lettuce and pea) were maintained under the same conditions as during propagation. Sporulation was assessed at 7 dpi for *Hpa* and *Bl*, and at 8 dpi for *Pvp*.

For conidiospore quantification, infected seedlings from each replicate were collected, with the roots discarded. For *Hpa*, 10 seedlings per replicate were placed into an Eppendorf tube containing 250 μL of SDW, while for *Bl*, three seedlings per replicate were placed into an Eppendorf tube containing 1 mL of SDW. For *Pvp*, three infected seedlings per replicate were placed into a Falcon tube containing 40 mL of SDW. The samples were gently vortexed to release the spores into suspension, then filtered through Miracloth. The conidiospores were collected by centrifugation and resuspended in 1 mL of SDW for assessment. Spore counts for all pathogens were performed by counting the number of spores present in all four corner grids of a haemocytometer. The sporulation percentage was calculated relative to the control group. Each experiment was repeated twice.

### 4.7 Multiplexing of SS‐dsRNA

Three *Hpa* genes, *HpaG802064*, *HpaG802452* and *HpaG803108*, were selected for the multiplexing experiments. SS‐dsRNAs targeting these genes were combined with the conidial suspension following the same protocol used for germination and sporulation assays. Instead of applying individual treatments, the SS‐dsRNAs were tested singly, in pairs, or as a triple combination, with each SS‐dsRNA in the mixture maintained at a final concentration of 5 μM. Each experiment was repeated twice.

### 4.8 Confocal Microscopy to Detect dsRNA Internalisation

To assess dsRNA uptake by *Hpa* spores, a 30‐nt SS‐dsRNA was designed based on *HpaG802307* (sense: 5′‐GUCUGAAAGGAGCCGAGAUUGACCUGAUUA‐3′; antisense: 5′‐UAAUCAGGUCAAUCUCGGCUCCUUUCAGAC‐3′) and synthesised with a Cy‐5 fluorophore attached to the 5′ end. *Hpa* or *Pvp* conidiospores were suspended in SDW at a concentration of 5 × 10^4^ spores/mL and mixed with 1 μM labelled SS‐dsRNA in an Eppendorf tube. The mixture was incubated on ice for 20 min to facilitate interaction between the spores and the dsRNA.

After incubation, 30 μL of the spore‐dsRNA mixture was pipetted onto microscope cavity glass slides. A control group containing spores without SS‐dsRNA was prepared similarly. The slides were placed in Petri dishes, covered and incubated under the conditions specified in the germination assay. Spores were examined 6 h post‐incubation using a Nikon Ti2‐E AXE NSPARC laser point‐scanning confocal system.

### 4.9 Isolation of Total RNA

Initially, we optimised the timing for RNA isolation for each pathosystem to perform gene expression analysis. For all three pathogens, 5 × 10^4^ spores/mL were used for inoculations. Gene expression was analysed using a combination of in vitro and in planta assays. For *BTUB* gene expression analysis in *BI* and *Pvp*, spores were mixed with 5 μM siRNA in a 1.5 μL Eppendorf tube and incubated under a 12 h light/12 h dark cycle at 16°C for 24 h. After incubation, the mixture was centrifuged at 12,000 *g* for 5 min, and the resulting pellet, containing conidiospores, was used for total RNA extraction. For *BTUB* and *CesA3* gene expression analysis in *Hpa*, and *CesA3* in *Bl* and *Pvp*, 5 μM dsRNAs were used for *Hpa*, while 10 μM were used for *Pvp* and *Bl*. For RNA extraction, 4‐week‐old *Arabidopsis* plants were used. A total of six leaves were marked for each plant, and each leaf was drop inoculated with 20 μL *Hpa* spore suspension with or without dsRNA. At 3 dpi, two leaves representing a biological replicate were collected in an Eppendorf tube and snap‐frozen in liquid nitrogen before storage at −80°C until RNA extraction. Similarly, lettuce and pea plants were inoculated with respective pathogen spore suspensions with or without dsRNA as described for the sporulation assay, and leaves were collected at 4 dpi and kept at −80°C until RNA isolation. Three biological replicates were set up for each treatment. Total RNA was extracted by RNeasy Plant Mini Kit (Qiagen), Zymo Quick‐RNA Plant Miniprep Kit (R2024) and Bioline Isolate II RNA Plant Kit (BIO‐52076) according to the manufacturer's instructions. RNA concentration and quality were evaluated by NanoDrop 2000c spectrophotometer (Thermo Fisher).

### 4.10 RT‐qPCR Analysis

To determine whether targeted genes were silenced by the application of dsRNA, RT‐qPCR was performed using Superscript III Platinum SYBR Green One‐Step qRT‐PCR Kit (Thermo Fisher). Primer sets used for amplification are listed in Table [Link], [Link]. Reactions were performed on Roche Light Cycler 480 II PCR machine using the following touchdown cycling conditions: initial incubation at 45°C for 10 min, followed by 40 cycles of denaturation at 95°C for 5 s, annealing starting at 68°C for 10 s (with the annealing temperature decreasing by 0.8°C per cycle from 68°C to 60°C over the first 10 cycles), and extension at 72°C for 5 s. Each RT‐qPCR contained 5 μL of SYBR Green PCR Master Mix, 0.3 μL of each 10 μM primer, 0.1 μL of reverse transcriptase, 0.2 μL RNA‐inhibitor, 1 μL RNA (at 70 ng/μL) and DEPC‐treated water for a final reaction volume of 10 μL. Melt curve analysis was performed to confirm amplification specificity. Data analysis was performed using the 2^−∆∆*C*t^ method, with gene expression normalised to the *actin* gene of the respective pathogen (*Hpa*, *Pvp or Bl*). For each sample, the mean of three replicates was used to calculate the relative change in mRNA levels.

### 4.11 Longevity Test

Seven‐day‐old *Arabidopsis* seedlings were drop inoculated using the same procedure as described for the sporulation assay, with the exception that additional seedlings were prepared for each time point. For each of the three biological replicates, samples were collected at 4, 7, 10 and 11 dpi. Seedlings were immediately placed into Eppendorf tubes, flash‐frozen in liquid nitrogen, and stored at −80°C until RNA extraction.

### 4.12 Bioinformatics

Primers were designed either using the Primer3 web tool (https://primer3.ut.ee) or using Geneious (v10.0) (Kearse et al. 2012). We used the EnsemblProtist (Kersey et al. 2016) and InterPro (Quevillon et al. 2005) databases to identify candidate *Hpa*‐*BTUB* gene. Reciprocal BLASTN and BLASTX (Altschul et al. 1997) were used to perform similarity searches of nucleotide and amino acid sequences using default settings, respectively, between *Hpa*, *Pvp and Bl* genes. Multiple sequence alignments were generated using MUSCLE v 3.8.31 (Edgar 2004) and visualised using Jalview v. 2.11.4.1 (Waterhouse et al. 2009).

### 4.13 Statistical Analysis

All statistical analyses and graph generation were performed using GraphPad Prism (v. 10.5.0 for Mac; GraphPad Software). Comparisons between treatments (e.g., 1 μM vs. 5 μM SS‐dsRNA) were conducted using unpaired two‐tailed *t* ‐tests. Where applicable, two‐way ANOVA was used to compare SS‐dsRNA‐treated and control samples, with Sidak's correction applied for multiple comparisons. Statistical significance was defined as ns = not significant, *p < 0.05, **p < 0.01, ***p < 0.001, ****p < 0.0001. Data are presented as means ± standard error of the mean (SEM) or standard deviation (SD), based on three independent biological replicates, as indicated. No post hoc tests were applied to *t* test comparisons.

## Author Contributions

Mahmut Tör and John M. McDowell conceived the research idea and designed the experiments with Deniz Göl. Deniz Göl, Emeka Okechukwu, Gizem Ünal and Sherif M. Sherif performed lab‐based experiments. Anne Webb, Theresa Wacker and David J. Studholme provided bioinformatic support while Tom Wood provided plant pathology support. Mahmut Tör, Yiguo Hong, Theresa Wacker, David J. Studholme, John M. McDowell and Deniz Göl analysed the data. All authors contributed to the writing and review of the manuscript and approved the final version for submission.

## Conflicts of Interest

The authors declare no conflicts of interest.

## Supporting information


**Figure S1:** mpp70140‐sup‐0001‐FigureS1.pdf.

**Figure S2:** mpp70140‐sup‐0002‐FigureS2.pdf.

**Figure S3:** mpp70140‐sup‐0003‐FigureS3.tiff.

**Figure S4:** mpp70140‐sup‐0004‐FigureS4.tiff.

**Table S1:** mpp70140‐sup‐0005‐TableS1.xlsx.

**Table S2:** mpp70140‐sup‐0006‐TableS2.xlsx.

**Table S3:** mpp70140‐sup‐0007‐TableS3.xlsx.

## Data Availability

The data that support the findings of this study are available from the corresponding author on reasonable request. Coding sequences of *Pvp‐CesA3* and *Pvp‐BTUB* can be accessed in GenBank under the accession numbers PV324775 and PV324774, respectively.
