# Supplementary material for: RNAi Strategies Against Downy Mildews: Insights Into dsRNA Uptake and Silencing
Source: Mol Plant Pathol. 2025 Aug 18;26(8):e70140. doi: 10.1111/mpp.70140 (PMC12358739; doi:10.1111/mpp.70140)
Supplement: Supplementary file 1 — Figure S1: mpp70140‐sup‐0001‐FigureS1.pdf. [file MPP-26-e70140-s005.pdf]

Launch in Jalview | View raw BioJSON Data

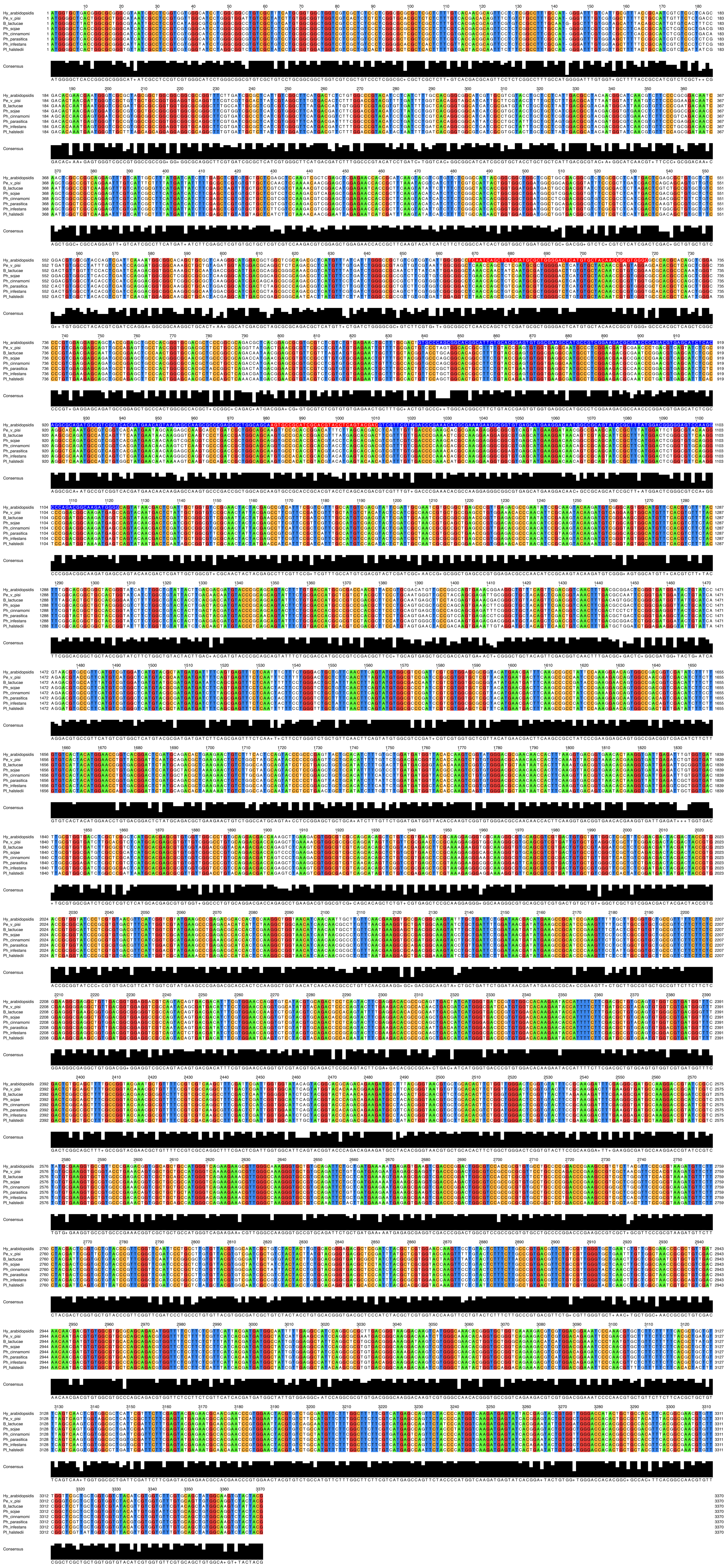

Supplemental Figure 1. Multiple sequence alignment of *CesA3* genes from oomycetes and model organisms. The black shading indicates the location of the sequence matching 30 bp long *Hpa-CesA3* Common-SS-dsRNA on the *Hyaloperonospora arabidopsidis* sequence. The blue shading indicates CesA3-*E. coli*-produced dsRNA. The black shading indicates *Hpa-CesA3* Specific SS-dsRNA. Sequences were aligned using MUSCLE v3.8.31. The accession numbers and sequence coordinates of the aligned oomycete sequences are:

- *Hyaloperonospora arabidopsidis* Emoy2 (Hy_arabidopsidis) HpaG810051
- *Pernonospora viciae* f. sp. pisi (Pe_v_pisi) this study
- *Phytophthora sojae* (Ph_sojae) RefSeq:XM_009535833.1
- *Phytophthora parasitica* (Ph_parasitica) RefSeq:XM_008915779.1
- *Phytophthora infestans* (Ph_infestans) RefSeq:XM_002897169.1
- *Phytophthora cinnamomi* (Ph_cinnamomi) RefSeq:XM_067930250.1
- *Bremia lactucae* (B_lactucae) RefSeq:XM_067963730.1
- *Plasmopara halstedii* (Pl_halstedii) RefSeq:XM_024717371.1

The alignment has been trimmed at the 3’-end.
