# Supplementary material for: RNAi Strategies Against Downy Mildews: Insights Into dsRNA Uptake and Silencing
Source: Mol Plant Pathol. 2025 Aug 18;26(8):e70140. doi: 10.1111/mpp.70140 (PMC12358739; doi:10.1111/mpp.70140)
Supplement: Supplementary file 2 — Figure S2: mpp70140‐sup‐0002‐FigureS2.pdf. [file MPP-26-e70140-s003.pdf]

Launch in Jalview   View raw BioJSON Data

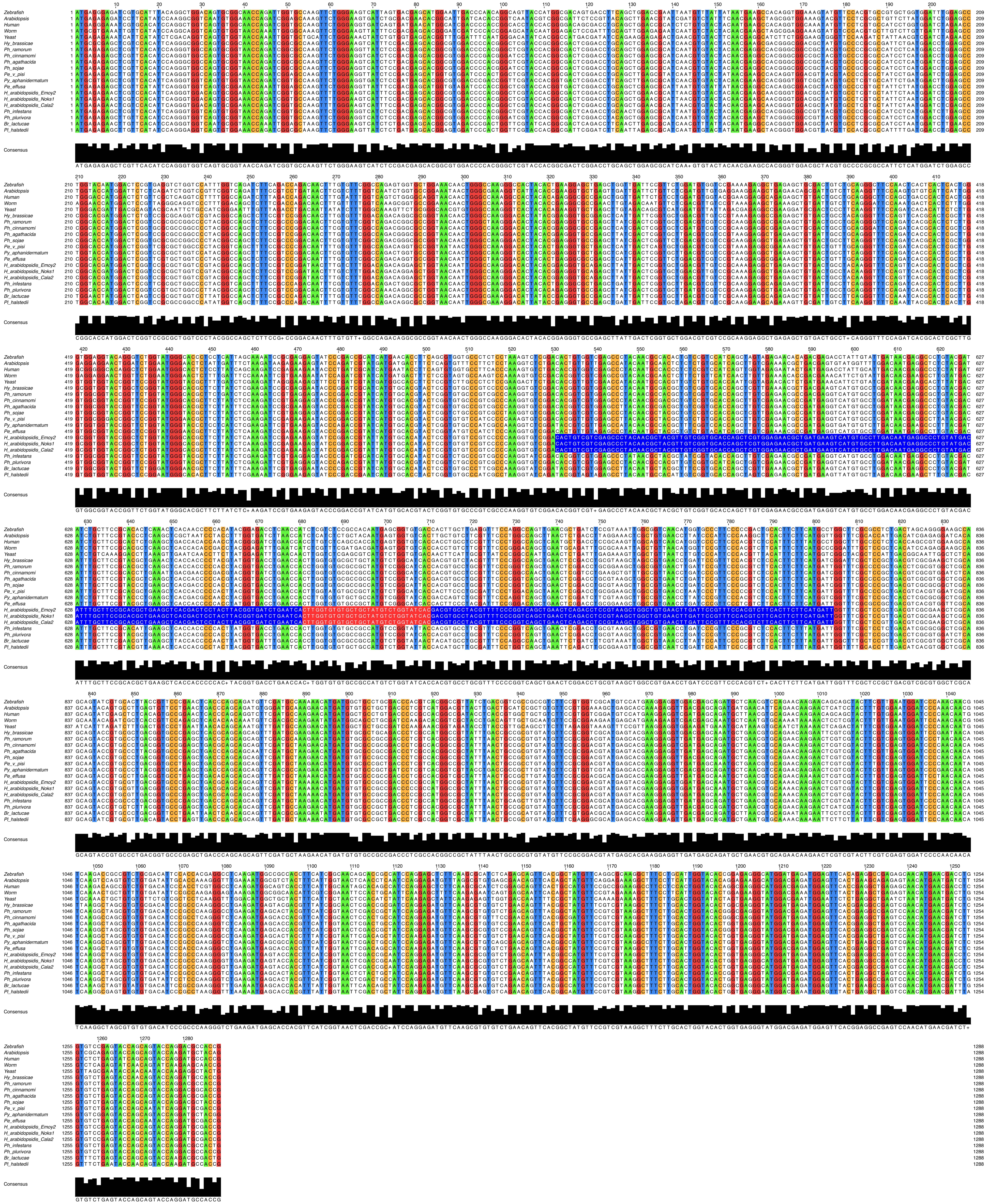

Supplemental Figure 2. Multiple sequence alignment of *tubulin* genes from oomycetes and model organisms. The red shading indicates the location of the sequence matching *Hpa-BTUB* SS-dsRNA. The blue shading indicates the location of the sequence matching *Hpa-BTUB E. coli*- produced dsRNA. The accession numbers and sequence coordinates of the aligned oomycete sequences are:

- *Bremia lactucae* (Br_lactucae) RefSeq:XM_067962673.1 (1-1290)
- *Hyaloperonospora arabidopsidis* Cala2 (H_arabidopsidis_Cala2) GenBank:LKIA01000455.1 (10041-11330)
- *Hyaloperonospora arabidopsidis* Emoy2 (H_arabidopsidis_Emoy2) GenBank:ABWE02003170.1 (1637-2926)
- *Hyaloperonospora arabidopsidis* Noks1 (H_arabidopsidis_Noks1) GenBank:LLKM01000460.1 (23019-21730)
- *Hyaloperonospora brassicae* (Hy_brassicae) GenBank:SZZI01000768.1 (16911-15622)
- *Pernonospora viciae* f. sp. Pisi (Pe_v_pisi) This study
- *Peronospora effusa* (Pe_effusa) GenBank:CP090803.1 (3022079-3023368)
- *Phytophthora agathidicida* (Ph_agathacida) GenBank:CP106975.1 (4903626-4904915)
- *Phytophthora cinnamomi* (Ph_cinnamomi) GenBank:U22050.1 (280-1569)
- *Phytophthora infestans* (Ph_infestans) RefSeq:XM_002908737.1 (87-1376)
- *Phytophthora plurivora* (Ph_plurivora) GenBank:CP125268.1 (4846179-4847468)
- *Phytophthora ramorum* (Ph_ramorum) RefSeq:XM_067894435.1 (109-1398)
- *Phytophthora sojae* (Ph_sojae) GenBank:CP155031.1 (1172078-1173367)
- *Plasmopara halstedii* (Pl_halstedii) RefSeq:XM_024718186.1 (202-1490)
- *Pythium aphanidermatum* (Py_aphanidermatum) GenBank:MK752999.1 (1-1290)

The accession numbers and sequence coordinates of the aligned model-organism sequences are:

- *Arabidopsis thaliana* RefSeq:NM_123801.2 (157-1445)
- *Caenorhabditis elegans* (Worm) RefSeq:NM_077184.8 (3-1291)
- Human RefSeq:NM_178014.4 (156-1445)
- *Saccharomyces cerevisiae* (Yeast) GenBank:CP029160.1 (1556403-1557690)
- Zebrafish RefSeq: NM_198809.2 (112-1400).

The alignment has been trimmed at the 3'-end.
