# Supplementary figures and images for: RNAi Strategies Against Downy Mildews: Insights Into dsRNA Uptake and Silencing
Source: Mol Plant Pathol. 2025 Aug 18;26(8):e70140. doi: 10.1111/mpp.70140 (PMC12358739; doi:10.1111/mpp.70140)

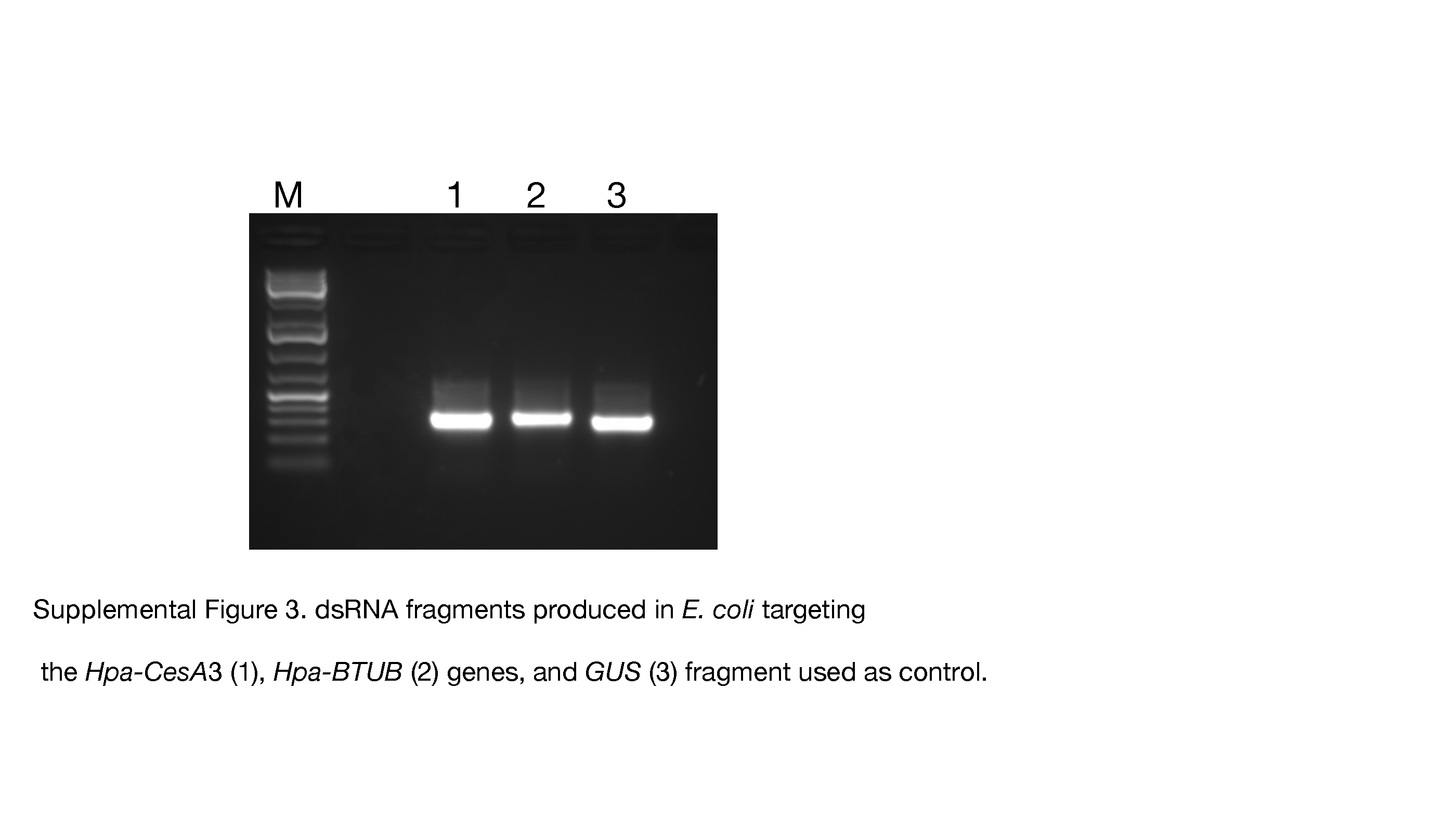

Supplement: Supplementary file 3 — Figure S3: mpp70140‐sup‐0003‐FigureS3.tiff. [file MPP-26-e70140-s002.tiff]

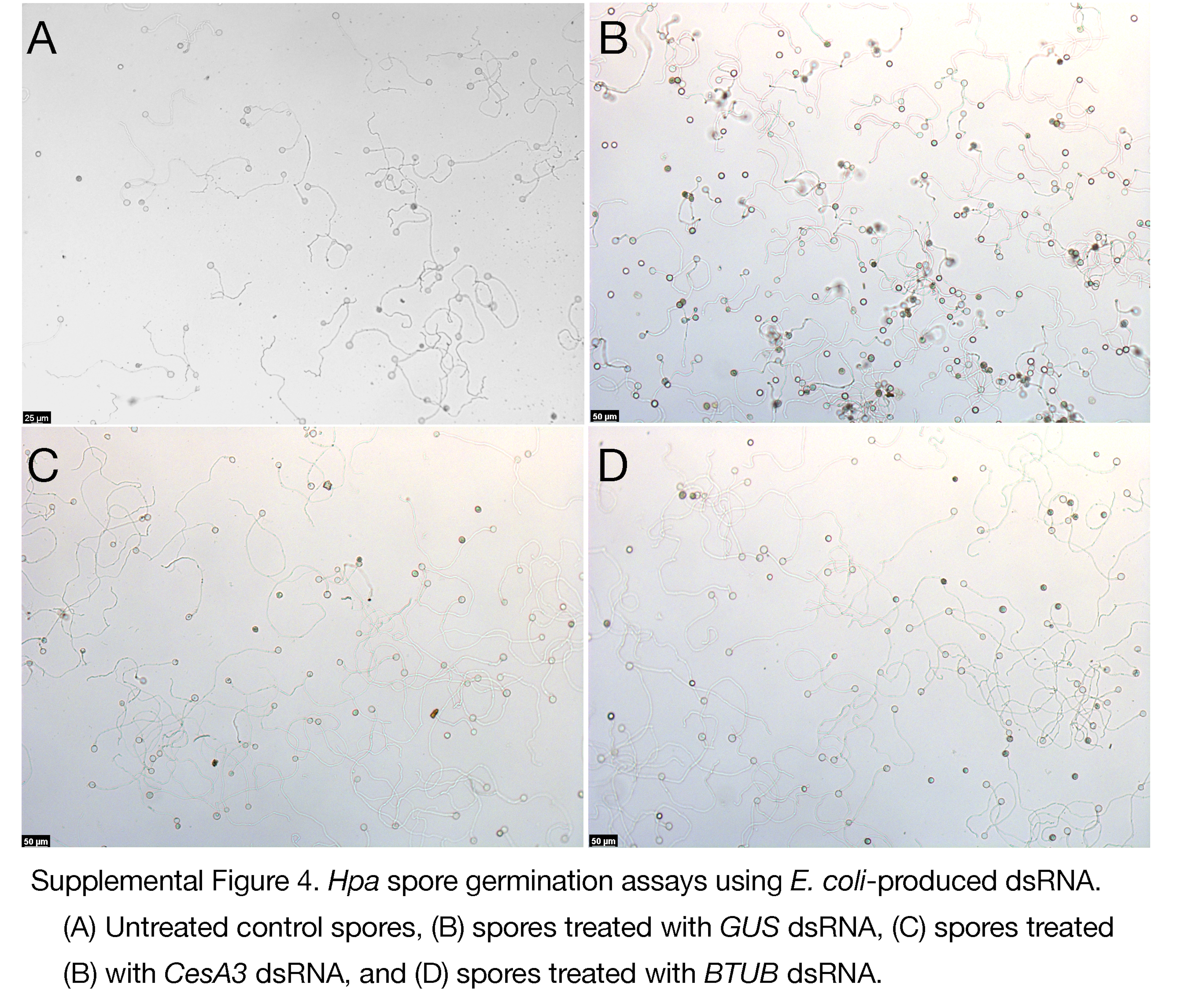

Supplement: Supplementary file 4 — Figure S4: mpp70140‐sup‐0004‐FigureS4.tiff. [file MPP-26-e70140-s004.tiff]
